# Differences in the Binding Affinities of ErbB Family: Heterogeneity in the Prediction of Resistance Mutants

**DOI:** 10.1371/journal.pone.0077054

**Published:** 2013-10-23

**Authors:** Mariana Pereira, Chandra S. Verma, Gloria Fuentes

**Affiliations:** 1 Bioinformatics Institute (Agency for Science, Technology and Research), Singapore; 2 Department of Biological Sciences, National University of Singapore, Singapore; 3 School of Biological Sciences, Nanyang Technological University, Singapore; Wake Forest University, United States of America

## Abstract

The pressure exerted by drugs targeted to a protein in any therapy inevitably leads to the emergence of drug resistance. One major mechanism of resistance involves the mutation of key residues in the target protein. Drugs that competitively replace a natural substrate are often made ineffective by mutations that reduce the drug’s affinity relative to that of the natural substrate. Hence atomic level understanding of the mechanisms that underlie this behavior is of utmost importance in efforts to design new drugs that can target such mutant proteins. Methods that can predict these mutations before they appear in clinic would be a major advance in the selection of the appropriate treatment strategy in patients. The present computational approach aims to model this emergence in EGFR and ErbB2 after treatment with the drug lapatinib, by investigating the structural, dynamic and energetic effects on these kinases when bound to the natural substrate ATP and to lapatinib. The study reveals binding modes and subpopulations that are presumably normally cryptic and these have been analyzed extensively here with respect to sites that are predicted to be hotspots for resisting mutations. These positions are compared in the context of currently available data from laboratory-based experiments and mechanistic details, at the atomistic level, of the origin of resistance are developed. The prediction of novel mutations, if validated by their emergence in the clinic, will make these methods as a powerful predictive tool which can be used in the design of new kinase inhibitors.

## Introduction

ErbB receptors are the prototypical members of the growth factor receptor tyrosine kinase (RTK) family. They are activated by the binding of a large array of different ligands that in turn induce receptor homo- or heterodimerization, autophosphorylation and consequently the propagation of signals from the extracellular space to the cytoplasm and nucleus of a cell. They orchestrate a number of biological processes, including normal cell proliferation, survival and differentiation [1], [2]. Aberrant activation of the ErbBs has been described mostly in relation to cancers [1], [3]. This deregulation can result from activating mutations, amplification and/or overexpression of EGFR or ErbB2 [4], [5]. The targeted inactivation of these disease-relevant kinases is often pursued with ATP competitive small-molecule tyrosine kinase inhibitors (TKIs) that block their enzymatic activity and thereby interfere with the phosphorylation of cellular substrates, thus blocking the propagation of the signal transduction. The 4-anilinoquinazoline class of tyrosine kinase inhibitors (TKIs) have been widely explored in different clinical phases [6], [7]. These include erlotinib which has shown satisfactory results in Phase III trials while gefitinib is now approved by the FDA. Lapatinib was approved as front-line combination therapy in ErbB2-positive breast cancer and as adjuvant in patients with disease progression after trastuzumab-based therapy [7]. Recent studies have shown EGFR and ErbB2 protein overexpression in a high percentage in lung cancer specimens. Therefore, patients with this type of tumor and high levels of these two tyrosine kinases may benefit from treatment with the dual inhibitor lapatinib [8] which has a therapeutic advantage over other inhibitors [9], including a slower rate of dissociation from EGFR and ErbB2, thus favoring extended downregulation of phosphorylation. From a structural perspective, although all these inhibitors are ATP competitive, they do target different kinase conformations: lapatinib binds to the inactive conformation, whereas erlotinib and gefitinib target the more active states of these kinases.

**Figure 1:**
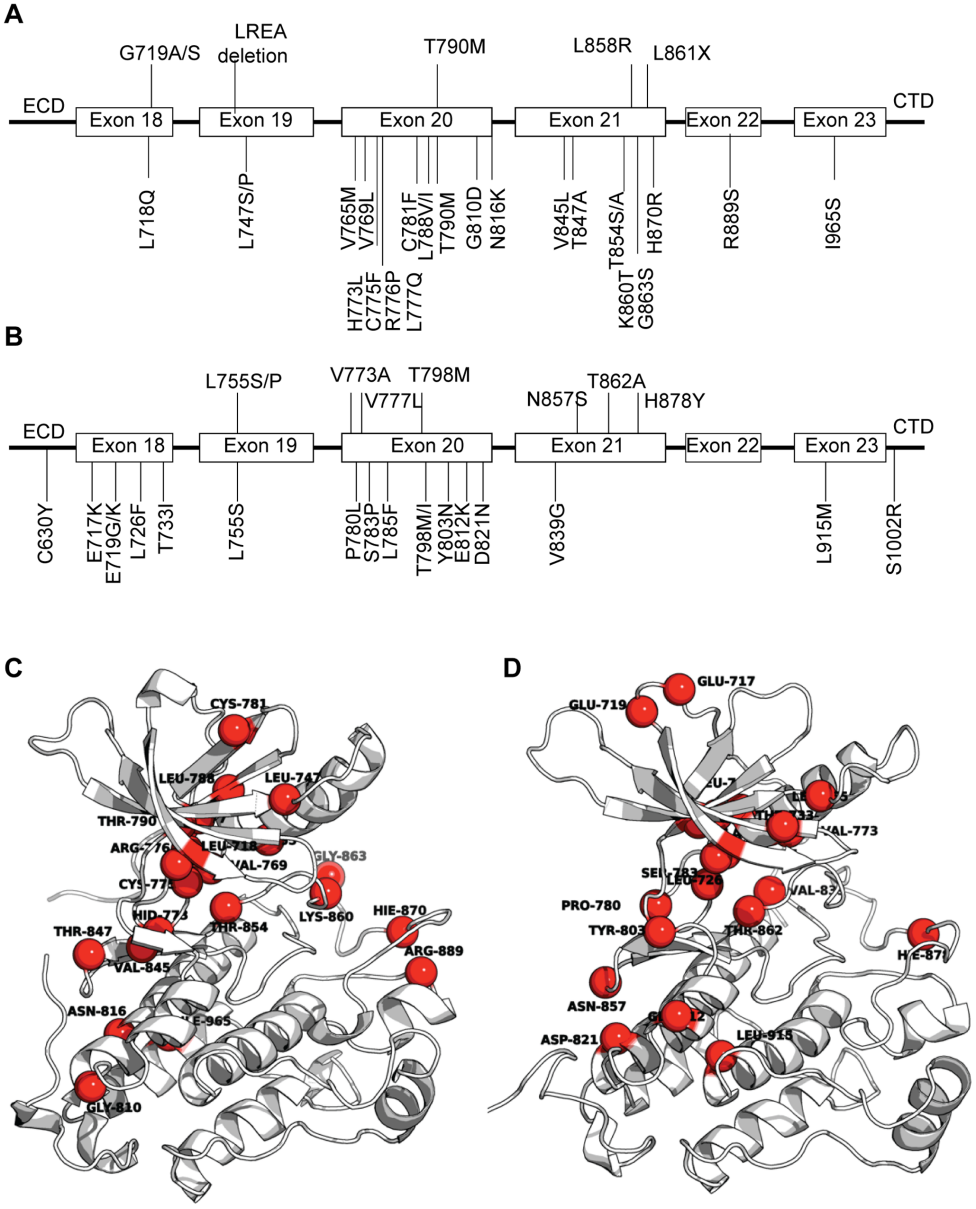
Literature reported mutations confering resistance towards lapatinib in EGFR and ErbB2. Genomic location of these mutations in the tyrosine kinase (TK) domain of EGFR (A) and ErbB2 (B). The list of mutations have been extracted from literature for both EGFR [16], [17] and for ErbB2 [18], [19]. These mutations have been mapped on the three-dimensional structures of EGFR (C) and ErbB2 (D).

In spite of the success of these molecules, they induce a strong selective pressure leading to resistance in their target tumor cells. This leads to abrogation of binding of these inhibitors, as a result of which these cells keep surviving and proliferating [10]. The outgrowth of a drug-resistant population represents an ongoing clinical problem. The mechanisms for conferring resistance have different origins [11], [12], including the amplification in kinase expression levels, rewiring of compensatory alternative signaling pathways [13] and/or most frequently, the accumulation of acquired secondary resistance mutations in the catalytic domains of the kinases [14]. Although no point mutations in EGFR or ErbB2 have been reported clinically as arising from lapatinib treatment yet, they eventually will, as has been seen for other kinase inhibitors; indeed they have been shown to emerge in laboratory-based mutational profiling studies. In these studies, lapatinib resistance in EGFR and ErbB2 has been seen to arise from mutations within residues that cluster at specific regions, notably encoding key structural features of the kinase, such as the “hinge” region, P-loop, activation loop, and in general the ATP-binding pocket. The location on the kinase fold of these mutations is such that they do not to compromise the catalytic activity of the kinase, but decrease the affinity for the inhibitors, thus recovering the catalytic activity of these kinases [15]. Several of the EGFR and ErbB2 variants [16]–[20] are mapped on the sequence and the 3D structure of both kinases in **Figure 1**. There is wide variation in the reports which possibly arises from differences in the experimental methods used to determine the affinities that these kinases have for the different inhibitors. Some studies have used *in vitro* substrate phosphorylation in a kinase assay, which is sensitive to the inhibitor binding to the fraction of active EGFR molecules; in contrast other observations are based on the changes in the intrinsic fluorescence of EGFR proteins induced by inhibitor addition, which depend on the compound binding to the total population of EGFR molecules in solution. The mechanism underpinning the effects of most of these mutations can be understood by examining the three dimensional structures of the kinases in complex with various ligands. However such analysis needs to be complemented with studies that reveal the dynamics that characterize these interactions, since structural studies cannot shed light on two key aspects: the role of dynamics in modulating the interactions and the role of mutations that are far from the active site whose effects need a more detailed computational analysis.

The computational work presented here aims to elucidate the mutations that might appear in EGFR and ErbB2 kinases in response to treatment with lapatinib. It complements experimental work by elaborating on the mechanistic details of the origin of the resistance at the atomistic level. Such approaches have the potential to be a powerful tool for the prediction of new mutations as well as aid in the development of new inhibitors that target these mutant strains. We carry out molecular dynamics simulations on the active and inactive states of EGFR and ErbB2 bound specifically to the natural ligand, defined here as one molecule of ATP, two magnesium ions and three water molecules, and to an inhibitor, comprised of a molecule of lapatinib and one water molecule. The interacting residues that contribute to the binding of lapatinib but have little contribution to the association with ATP have been identified using *in silico* alanine scanning. This study further adds to the study carried out by Rizzo and coworkers on predicting EGFR resistance towards gefinitib and erlotinib [8] and lapatinib [21] by using a recently developed tool that uniquely enables the analysis of the interactions with lapatinib that emerge from different conformational subpopulations. These findings are backed by the observations that a large number of the putative mutants predicted within this study have been reported in different laboratory-based experiments [16]–[20], lending a degree of confidence to our methodology.

## Results

### Simulation Sampling and Stability

Molecular Dynamics simulations have been performed in order to analyze the dynamics and interactions of EGFR and ErbB2 with their natural ligand ATP and with an inhibitor lapatinib (FMM). For each system, we have carried out three 50 ns MD simulations in order to ensure that the events and populations observed were reproducible, at least qualitatively. The different trajectories have been assessed for stability using the measure of the temporal evolution of structural root-mean-square-deviation (RMSD). The RMSD values for the proteins (both EGFR and ErbB2 kinase domain) obtained after fitting the Cα atoms of each snapshot to the first frame of the trajectory are shown in Figure S1. It is clear that the simulations are stable. For all the systems, the trajectories stabilize around 10–20 ns, confirming that the systems have reached equilibrium. We believe that the 600 ns obtained with the simulations in this work provide enough sampling for the systems under investigation. Furthermore, the time evolution of the secondary structure for the four systems have been reported as an additional parameter to assess the stability of the kinase fold during the length of the simulations (Figure S4).

### Binding Energy: The Effect of Different Populations in the Conformational Spaces of the Bound Systems

**Figure 2:**
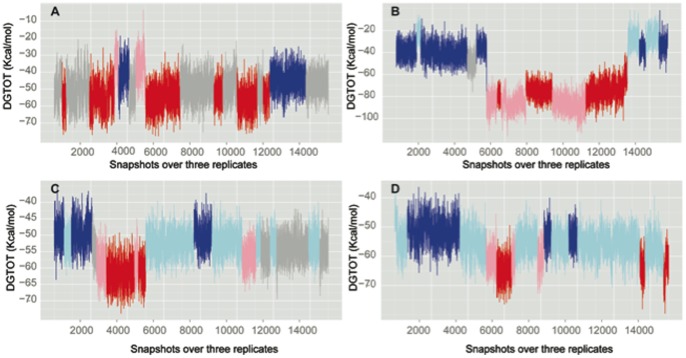
Time series for the total binding energy for EGFR in association with ATP (A) and lapatinib (C), and the same inhibitor-protein systems for ErbB2, panels B for the interaction with ATP and D for the lapatinib one.

The time series of the binding free energies, calculated using a Molecular Mechanics-Generalized Born Solvent Accessibility MM-GBSA approach (Figure 2) show large fluctuations, which suggest the co-existence of diverse populations. These could arise from events such as reorganization of water molecules within the binding pocket or from the ligand exploring different conformational spaces. In order to identify and define different populations in the binding modes of ATP and lapatinib to these kinases, we have used the MMPBSA_segmentation method previously developed in our group [22]. This method enables the treatment of the time series of the computed binding free energies as arising from multiple separate segments, each of which represents a conformational substate. The advantage of this technique is that in contrast to averaging over a time series and extracting a mean, which will necessarily represent the conformation sampled most, this new method enables the extraction and analysis of more diverse conformations, including those that may be short-lived. The identification of short-lived modes may offer advantages in the design of specific inhibitors. In the case of EGFR bound to ATP in coordination with ions and waters (Figure 2A), there is a larger subpopulation (shown in gray) with a mean value of −56.3 kcal/mol representing a looser binding mode and a slightly less populated state characterized by stronger binding (mean value −62.3 kcal/mol, shown in red) (Table 1). In addition, we find a sparse looser subpopulation (shown in blue) where the difference with the tight binding population is ∼12 kcal/mol. In ErbB2 (Figure 2B), the configurational space sampled in our simulations appears to be more extensive, covering more distant regions of the binding landscape (Figure S2). For this enzyme, the differences in the looser (shown in blue) and tighter (shown in red) binding modes are more apparent, with a difference of ∼36 kcal/mol. One of the replicates extensively explores the looser conformation, while another replicate preferentially explores the tighter binding mode, and the third replicate appears to oscillate between the two modes thus suggesting that it is likely that within the timescale of these simulations, the system resides within these two modes.

**Table 1:** Individual energy components for the estimated binding energies for the interaction of ATP and lapatinib with EGFR and ErbB2 calculated with MM-GBSA method.

		%occ	<ΔE^vdw^_int_>	<ΔE^ele^_int_>	<ΔG^polar^_sol_>	<ΔG^nonpolar^_sol_>	<ΔG_bind_>
EGFRa-ATP.2MG.3KOK	gray	41.5	−28.0±7.6	−393.7±23.9	372.8±18.7	−7.3±0.3	−56.3±7.7
	red	36.4	−31.0±8.5	−394.3±22.9	370.3±17.9	−7.3±0.2	−62.3±7.6
	pink	5.2	−23.3±6.0	−357.6±22.8	343.9±15.2	−7.1±0.1	−44.2±8.5
	blue	16.9	−27.7±7.7	−372.5±18.4	356.4±14.5	−7.1±0.3	−50.9±6.7
ErbB2a-ATP.2MG.3KOK	blue	33.9	−21.2±6.2	−302.0±42.3	285.0±40.4	−6.3±0.2	−44.5±7.1
	lightblue	10.8	−16.5±6.4	−348.9±53.5	341.0±46.0	−6.1±0.2	−30.5±8.2
	gray	3.4	−22.5±6.1	−305.4±14.2	279.9±10.2	−6.5±0.1	−54.5±7.7
	pink	25.5	−20.5±5.8	−444.9±19.3	387.4±15.2	−7.1±0.4	−94.1±7.5
	red	26.4	−19.1±6.4	−420.5±19.5	364.7±16.2	−7.0±0.3	−81.9±8.0
EGFRi-FMM.1WAT	blue	17.6	−74.6±3.5	−34.5±6.9	59.3±5.8	−9.2±0.3	−59.1±3.9
	lightblue	39.4	−74.8±4.0	−38.1±5.8	60.2±5.3	−9.1±0.4	−61.9±3.6
	gray	19.8	−75.1±3.9	−38.4±5.0	59.0±4.9	−9.0±0.4	−63.5±3.5
	pink	10	−84.2±3.6	−40.5±6.1	65.9±4.0	−9.6±0.1	−68.4±4.2
	red	12.9	−85.7±2.8	−43.9±5.2	68.2±3.6	−9.5±0.1	−71.0±3.9
ErbB2i-FMM.1WAT	lightblue	58.4	−75.7±3.5	−39.8±8.2	60.5±6.8	−9.3±0.5	−64.2±4.1
	blue	24.9	−75.6±3.0	−34.3±5.3	56.8±5.4	−8.8±0.3	−61.9±2.8
	pink	7.6	−75.5±3.2	−40.6±8.6	54.9±7.5	−8.6±0.3	−69.8±4.2
	red	9.0	−78.3±2.8	−46.0±4.7	62.9±4.1	−8.7±0.1	−74.1±3.6

The values represent the average and standard deviation for the different populations observed in Figure 2. The values are given in kcal/mol.

For the case of the inactive kinases bound to lapatinib and a water molecule (see below), once again we observe the existence of different subpopulations. For both EGFR (Figure 2C) and ErbB2 (Figure 2D), two extreme ensembles are identified, with some occupied subspaces in between. The difference in energy between the tighter (in red) and looser (in blue) modes is around ∼12–13 kcal/mol.

The presence of these “so-called subpopulations” separated by the large energies point at the existence of distinct binding modes/poses. We next characterize the structures and dynamics underlying these poses in order to assign them to relevant macrostates.

### Affinity of EGFR and ErbB2 Towards ATP and Lapatinib

The binding energies for ATP and lapatinib with EGFR and ErbB2 in the different subpopulations have been estimated using MM-GBSA calculations after grouping the stretches of the trajectories identified. In the case of the ATP bound systems, we have included in these calculations two Mg2+ ions and three water molecules that are hypothesized to be biologically necessary for the catalytic function. For some of the simulations carried out here, there has been a replacement of one of these water molecules with a water molecule from the bulk solvent, however all the systems behave consistently with three key water molecules always present in the catalytic site. For the lapatinib bound cases, we observed the constant presence of a water molecule in the binding proximity of the inhibitor. This water was observed despite its absence in the initial structure, and hence we deem this to be structurally important and we have included it in all the calculations. Indeed the original structure solved by crystallography (PDB id: 1×kk) has a water molecule located at a very similar position.

The different contributions to the binding energy for all the four systems are shown in Table 1. These values represent the averages for the different populations observed in Figure 2 and we believe that they represent macrostates. For the ATP bound systems, the driving force differentiating the subpopulations comes from the polar component, with ∼73 kcal/mol separating the extreme subpopulations. It is clear from Table 1 that despite the high desolvation penalty associated with embedding the ATP together with the ions and water molecules in the catalytic site, the binding is driven by a large electrostatic stabilization induced by the interactions with residues in the kinase together with lesser contributions from hydrophobic interactions. In the case of hydrophobic lapatinib, understandably the desolvation penalty is much smaller (around 80% of the surface is non-polar), and binding is driven by the van der Waals contribution arising from hydrophobic contacts that lapatinib makes with an extensive network of residues located within certain regions in the catalytic pocket. Consequently, the difference in the subpopulations arises from the van der Waals term; for example in the case of EGFR bound to lapatinib, the differences between the tighter and looser binding modes arises from ∼2 kcal/mol and ∼11 kcal/mol polar and nonpolar contributions respectively.

### In silico Alanine Scanning Mutagenesis: What is the Contribution of each Residue in the Different Binding Modes in the Four Systems?

**Figure 3:**
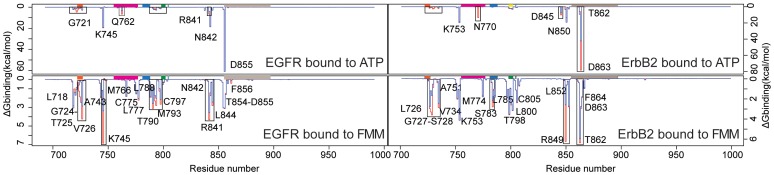
Residue-wise contribution estimated by *in silico* alanine scanning for the systems analysed in this study (Table S1). The coloured boxes on the top part of the plots represent key secondary structure elements in the kinase architecture (see Figure 4A). The graphs in red correspond to the tighter binding mode while the blue ones are for the identified subpopulations with a lesser interaction or looser binding mode. The regions showing a distinct binding energy for these two modes have been marked with boxes.

We have estimated the contribution for each residue (“binding affinity hotspots”) in the binding with the two ligands for the different subpopulations identified, using computational alanine scanning by mutating every single residue in the kinases and then carrying out single point GB energy calculations. This method assumes that the replacement of a residue by alanine will only perturb the local environment and hence not alter the binding mode. Figure 3 shows the residue-wise contribution profiles for the tighter and looser binding modes. For the active states (top panels), the interaction profile reveals a very localized set of residues involved in the binding with a high energetic contribution, arising mainly from the electrostatic contributions. All these residues make hydrogen bonds with the phosphate tail of ATP and with the water molecules, and contribute to the stabilization of the Mg2+ coordination spheres (see Table 2; for a whole description of the interactions please see Table S2, S3, S4, S5). While most of the residues that contribute significantly to the interactions in each mode are similar in identity (but vary in the amplitude of their contributions; see the profile in red with respect to blue in the top panel of Figure 3), certain residues do seem to be characteristic of a particular binding mode (e.g. Gln770 in ErbB2 is only implicated in the tighter mode).

**Table 2:** Molecular interactions monitored during the simulations of EGFR and ErbB2 bound to ATP and lapatinib.

System							
EGFR	ATP@O2γ		ATP@O3γ	WAT1@O	WAT2@O	Asp855@Oδ1	Asp855@Oδ2
	ATP@O2γ		ATP@O3γ	WAT3@O	Asn842@Oδ1	Asp855@Oδ1	Asp855@Oδ2
ErbB2	ATP@O2γ		ATP@O3γ	WAT1@O	WAT2@O	Asp863@Oδ2	Asp863@Oδ2
	ATP (O2γ)		ATP@O3γ	WAT3@O	Asn850@Oδ1	Asp863@Oδ1	Asp863@Oδ2
**Hydrogen bonds detected in the active kinases bound to the natural ligand ATP.2MG.3WAT.**							
	**phosphate tail**		**sugar moiety**	**adenosine ring**			**water molecules**
EGFR	Lys745@Nζ, Arg841@Nη1, Nη2			Thr790@Oγ1, Gln791@O			Lys745@Nζ, Glu762@Oε1, Arg841@O, Asn842@Oδ1 Asp855@Oδ1,Oδ2
ErbB2	Lys753@Nζ, Arg849Nη2,Nη1, Asn850@Nδ2, Thr862@Oγ1		Lys753@Nζ	Thr798@Oγ1, Gln799@O			Glu770@Oε1,Oε2; Asp845@Oδ1,Oδ2; Arg849@O,Nη2; Asn850@Oδ1;Asp863@Oδ1
**Specific hydrogen bonds found in the tighter and looser binding modes for ErbB2 in complex with ATP.**							
**ErbB2 residue**				**Interaction in looser binding mode**		**Interaction in tighter binding mode**	
Glu770@Oε1						WAT1@O	
Lys753@Nζ				ATP@O5′		ATP@O1α	
Arg849@Nh1						ATP@ O3γ	
Asn850@Nδ2				ATP@O3γ			
Thr862@Oγ1				ATP@O1α			
Asp863@Oδ2				WAT1@O		MG2	
**Hydrogen bonds in the complexes of EGFR and ErbB2 with lapatinib and a water molecule.**							
**Lapatinib**		**EGFR**			**ErbB2**		
		**looser mode**		**tighter mode**	**looser mode**	**tighter mode**	
FMM@N7		Ser720@O, Leu718@O				Ser728@N	
FMM@N18		Met793@N Thr790@Oγ1		Met793@N	Met801@N	Met801@N	
FMM@N20				Thr790@Oγ1	Thr798@Oγ1	Thr798@Oγ1	
FMM@N22		Thr854@Og1…WAT		Thr854@Og1 … WAT	Thr798@Oγ1, WATThr862@Oγ1, WAT	Thr798@Oγ1…WATThr862@Oγ1…WATSer783@Oγ…WAT	
FMM@O4				Thr725@N, Val726@N		Ser728@Oγ,Arg849@Nη1	
FMM@O3				Lys745@Nζ			

**Figure 4:**
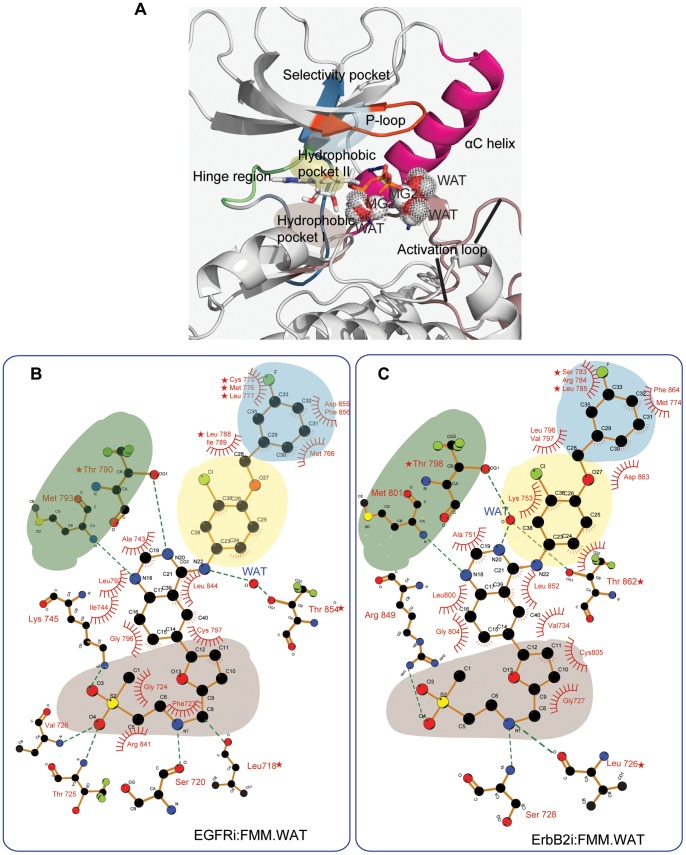
Pharmacophore architecture and description of the ATP binding pocket. ATP binds to a deep cleft situated between the N- and C-lobes of the protein kinase (A). In general, the ATP binding pocket can be divided into several regions that they form a continuos space with residues that belong to the hinge region (in green), P-loop (in orange), αC-helix (in pink) and activation loop (in brown). The selectivity pocket (in blue) and hydrophobic pockets I (in gray) and II (in yellow) are highlighted. Schematic 2D representation of the binding modes of lapatinib with EGFR (B) and ErbB2 (C) are depicted.

The binding of lapatinib appears to be determined by hydrophobic interactions with residues located at key regions in the ATP binding pocket (bottom plots in Figure 3) and a few hydrogen bonds (Table 2). In the case of EGFR, the gatekeeper Thr790 forms a direct hydrogen bond with lapatinib, while Thr854 is bridged to the drug through a water molecule; in contrast, in ErbB2, the equivalent Thr862 and Thr798 are both bridged with lapatinib through a water-mediated hydrogen bond (Figure 4B and 4C).

Another point to highlight is the similarity in the residue profile patterns and positions observed for EGFR and ErbB2 in both the active and inactive bound states (Figure 3). This suggests that similar resistance mutations would likely arise in both these proteins to lapatinib since the binding modes appear to be highly conserved which might make it easier to design inhibitors that work against both mutant enzymes.

### Dissecting the Individual Contributions of Residues in EGFR and ErbB2 Involved in the Binding of ATP and Lapatinib

Hydrogen bond and Mg2+ electrostatic interaction analysis for the active conformation of the kinases bound to ATP.

ATP binds to a deep cleft situated between the N- and C-lobes of the protein kinase. In general, the ATP binding pocket can be divided into several regions (Figure 4A) with residues that belong to the hinge region, P-loop, αC-helix and activation loop providing well-defined pharmacophores. The largest contribution in the binding of ATP comes from the electrostatic charge-charge interactions established by the Mg2+ ions with negatively charged residues from the two coordination spheres. The ionic interactions have been monitored during the entire simulation for all the trajectories (Table 2). These positions are occupied by the three key water molecules, the oxygen atoms at position **γ** of the ATP phosphate tail and a particular set of hydrophilic residues in the surrounding regions, being Asn842 and Asp855 in EGFR and equivalent Asn850 and Asp863 in ErbB2 (Figure S3). These data show that both magnesium ions remain hexacoordinated, although there are a few other residues that get loosely engaged in the stabilization of the sphere.

The hydrogen bonds formed between hydrophilic residues with the three water molecules and the oxygen atoms of the phosphate tail of ATP also provide a high contribution to the electrostatic stabilization of the system (Table 2). Residues engaging in hydrogen bonds with the phosphate tail of ATP are Lys745 and Arg841 in EGFR, while in the case of ErbB2 are the equivalent Lys753 and Arg849 and additionally Asn850 and Thr862 (Figure S3). These two new residues interacting in the binding with ErbB2 define one of the two binding modes we have extracted; Thr862 replaces Lys753 in the hydrogen bond with O1γ, while Asn850 does the same for Arg849 with the oxygen at position 3γ (Table 2).

**Figure 5:**
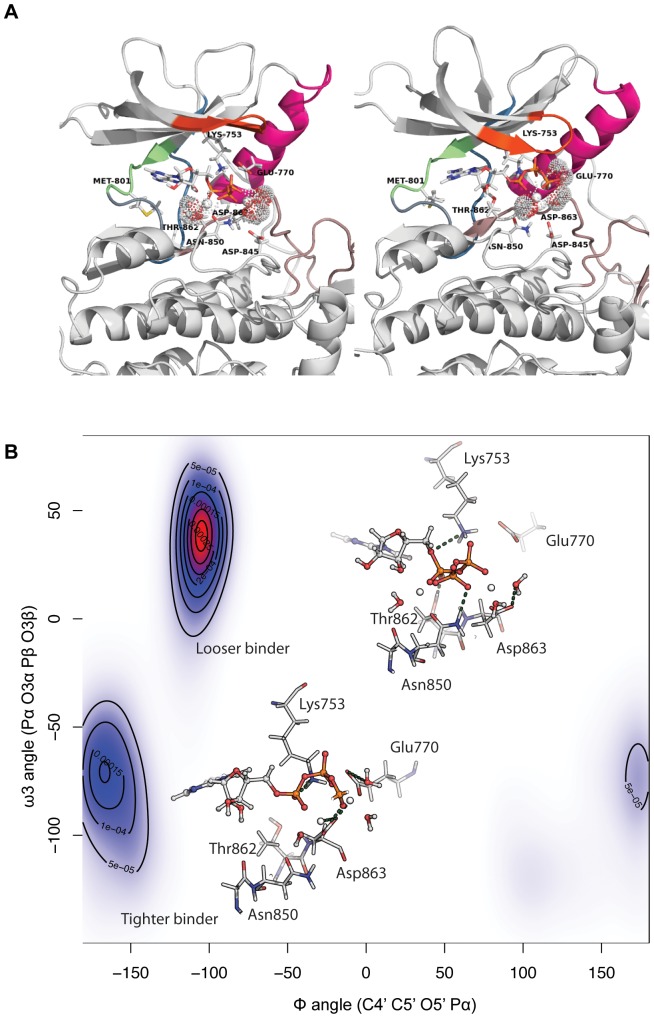
Different binding modes for ErbB2 bound to ATP. (A) Global kinase conformation for the looser (picture on the left) and the tighter (picture of right) binding modes. The color scheme is the same that described in Figure 4A) Two dimensional plot of the Φ and ω3 angles of ATP, where isolines represent the 2D kernel density estimation as calculated using an axis-aligned bivariate normal kernel evaluated on a square grid. Different rotameric states of ATP lead to a different interaction profile with neighbouring residues in the pocket.

The P-loop also shows a favorable binding energy, originating largely in van der Waals interactions between the hydrophobic carbons in the ATP molecule and a few amino acids in this region and these are conserved. Although the magnitude of the interactions is small, we observed a different interaction profile of the P-loop for the tighter binding mode with respect to the looser binding conformation (Figure 5A). Another region making hydrogen bonds with the adenosine ring of ATP lies in a completely different side of the pocket; it encompasses the gatekeeper Thr790/Thr798 and consecutive residues in the hinge region: Gln791/Gln799 for EGFR/ErbB2 respectively.

The relatively small number of residues implicated in the electrostatic interactions dominating the association of ATP in these systems points to conserved hydrophilic hotspots in the ErbB family. This association is with the natural ligand and suggests that these residues, or at least interactions of similar magnitude at these locations, in general cannot be compromised and hence are not under selective pressures to mutate; of course this will be validated only when the spectrum of mutations in the clinic after lapatinib treatment become available. Despite the remarkable similarity between the different binding modes for the systems with ATP, there are subtle variations between the tighter and looser conformations; for EGFR, Glu762 and Arg841 have a higher contribution in the tighter binding mode (see boxes in Figure 3). For ErbB2, the profile is even more distinct between the two modes with the tighter mode characterized by large electrostatic contributions from Glu770, Asn845, Arg849 and especially Asp863. Asp863 undergoes a conformational change from a direct engagement with the magnesium ion in the tighter mode to a water-bridged interaction with the magnesium ion in the looser mode (Table 2). In this particular case, the interaction of Glu770 is only present in the tighter binding mode. The differences observed arise from the different conformations that ATP adopts in these subpopulations, especially in the Φ and ω3 angles. In the tighter binder mode (Figure 5B), the α phosphate group is pushed upwards (considering the orientation depicted in Figure 4A), while the γ phosphate group moves in towards the interior of the pocket. These different orientations induce a reorientation of the Mg2+ ions and the three water molecules in such a way that Glu770 partially makes a new hydrogen bond with a water molecule, and Asp863 participates directly in the coordination sphere of a Mg2+ ion (in the looser binding mode, Asp863 bridges a water molecule). Another distinct interaction is the formation of a hydrogen bond between Lys753 and the linker oxygen between the sugar moiety and the ATP phosphate group in the looser mode, which is absent in the tighter mode. The disposition of the phosphate tail of ATP in the tighter mode is accompanied by a distortion in the orientation of the P-loop which now adopts two different orientations within the kinase pocket (Figure 5A). In the tighter binding mode, this β-hairpin, and especially the connecting β1 sheet, close up on top of the ATP molecule creating a more confined space (Figure 5A, picture on the right), thus creating the favorable interactions seen in the interacting profiles.

The van der Waals interactions arise from three different locations within the ATP-binding pocket: the top part, comprising of residues within and around the P-loop, which includes Leu718 to Val726 in EGFR and Leu726 to Val734 in ErbB2-; the bottom part, formed by residues Lys846 and Leu858 (for both EGFR/ErbB2); the walls of the pocket with Ile744, Pro794, Leu798 (for EGFR) with the corresponding positions in ErbB2 at Leu852, Thr862, Ala851, Ala751, Lys753, Leu800, Met801, and Cys805 (Tables S8 and S9).

### Hydrogen Bond and Hydrophobic Interaction Analysis for the Systems Bound to Lapatinib

In the case of lapatinib, there are very few hydrophilic interactions that contribute to the binding of the inhibitor (Table 2 and Table S6–S7). For EGFR, Thr790 and Met793 engage in direct hydrogen bonds with the quinazoline ring, while Thr854 interacts with the aniline N through a water molecule. For ErbB2, few residues in the hinge region make hydrogen bonds with the quinazoline ring: Met801 and gatekeeper Thr798 make direct hydrogen bonds while Thr798, Thr862 and transiently Ser783 are water-bridged to lapatinib. The P-loop region in both kinases hydrogen bonds with the solvent-exposed methylsulfonylethylamino moiety, in particular through Leu718, Ser720, Thr725, Val726 for EGFR, and Leu726 and Ser728 in ErbB2. In the case of ErbB2, Arg849 is involved in an interaction with the oxygen atom in this solvent-exposed moiety, being particularly strong in the tighter binding mode. In contrast in EGFR, Lys745 stabilizes the inhibitor. The water-mediated interactions are similar to those seen in the crystal structures of EGFR bound to erlotinib and gefitinib.

The van der Waals interactions are quite diffuse and account for around 65% of the total contributions to the binding energy of lapatinib as expected for a hydrophobic ligand (Table 1). The adenine and ribose pockets are somewhat hydrophobic, and it is in this particular cavity where the planar quinazoline ring is sandwiched mainly by Ala743/Ala751, Leu792/Leu800 and Ile744 on one side of the plane, and Leu844/Leu852 on the other for EFGR/ErbB2 respectively (Tables S10 and S11). Most of the nonpolar residues in the β-sheet preceding the P-loop,including Leu718/Leu726, Gly719/Gly727, Val926/Val734 for EGFR/ErbB2 constitute the top of this cavity, while the bottom is defined by Leu844/Leu852 and Cys797/Cys805 for EGFR/ErbB2 respectively. The 3′-chloro-4′-[(3- fluorobenzyl)oxy]aniline group makes predominantly hydrophobic interactions with the back of the ATP binding site which includes Leu788-Ile789, Met766 and Asp855-Phe856 (located in the DFG motif) for EGFR, and the equivalent ones for ErbB2 (Leu796, Val797, Met774, Asp863, Phe864 in Figure 4C). All these residues constitute the so-called allosteric or selective pocket where the 3-fluorobenzyloxy group sits (Figure 4A). The solvent exposed methylsulfonylethylaminomethylfuryl group has few hydrophobic contacts, as seen in the crystal structure.

**Figure 6:**
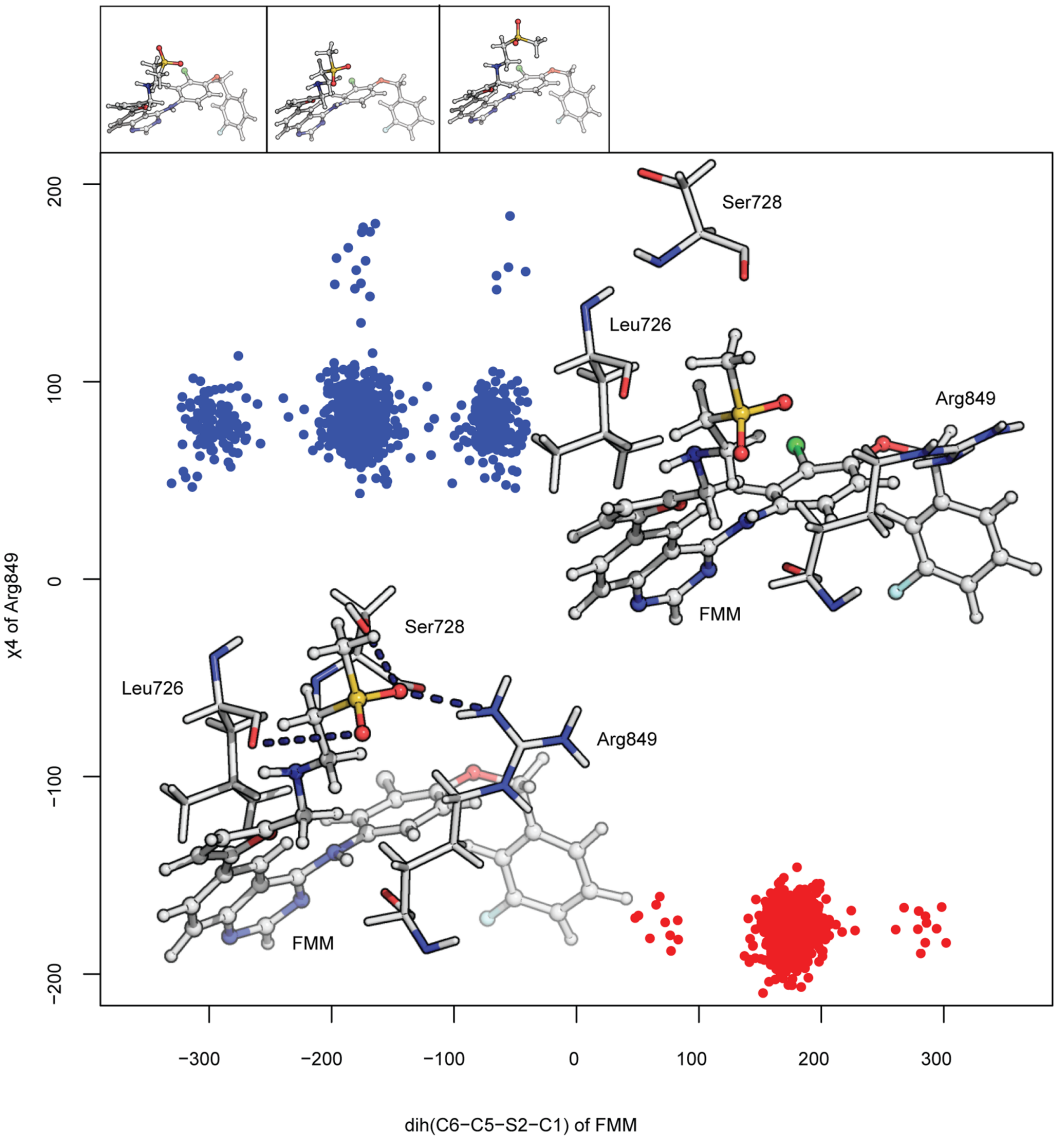
Different binding modes for ErbB2 bound to lapatinib. Two dimensional χ-angle plot for Arg849 (χ4) and the dihedral angle in lapatinib comprising the rotation along C5 and the sulphure atom at the methylsulfonyl group for the binder and looser conformations explored in the triplicate simulations for the system. Conformations are color-coded with respect to these two different populations and following the scheme in previous figures. Representative structures of Arg849 and Ser728 sidechains and their interactions with lapatinib are shown inside the plot, with hydrogen bonds indicated by dark blue dashed lines. Different conformers observed along the dihedral angle in lapatinib are shown on top of the plot; they are the equivalent to the so called gauche-, trans and gauche+ conformations; they correspond to the clusters near −60°, 180° and 60° in the main plot.

As we have seen for the active conformations of the two kinases, remarkably few differences are found when comparing the tighter and looser binding modes (Figure 3, bottom panels). Although the residues that contribute to this difference vary between ErbB2 and EGFR, the pattern follows a similar trend, with the main interactions located in the P-loop, hinge region and the region preceding the activation loop (pale gray). In the case of ErbB2, these distinct interactions arise from enhanced polar contributions of Gly727, Ser728, Ser783, Thr862 and especially Arg849, with a difference in the total energy of ∼3.5 kcal/mol. In order to characterize the structural features that result in the different contributions in the two binding modes, we have extracted the dihedral angles for the side chain of Arg849 together with the different lapatinib conformers in the two subpopulations. It is possible to find two descriptors that are the distinguishing feature between these modes; the χ4 dihedral angle for the arginine and the conformers around C5-S2 of lapatinib can be seen to separate them into two well defined groups (Figure 6). The most differentiating observable arises from the rotameric states that Arg849 shows in the two populations; in the case of the tighter binder this arginine adopts an energetically favorable trans conformation (∼180°) that favors the formation of a hydrogen bond with lapatinib. In the looser binding mode, the guanidino group at the side chain of arginine points towards the solvent and away from lapatinib, with an angle of around 80°, such that the electrostatic interaction with lapatinib is replaced by that with a water molecule from the solvent. The second observable is the C5-S2 dihedral in lapatinib which describes the tighter mode populating the region around *trans* with very few states exploring the *gauche* conformations, while the looser mode shows a larger variability in the orientation of the solvent exposed tail of lapatinib, with a nearly 50–50 probability of *trans* relative to either *gauche* conformation (Figure 6). This larger rotational space appears to be associated with the absence of electrostatic interactions with Ser728 adjacent to the P-loop, that anchors lapatinib uniquely in the tighter mode and there is indeed a correlation between the lapatinib conformation and the orientation of the β-hairpin in the P-region.

### Predicting Resistance Mutations in EGFR and ErbB2

**Figure 7:**
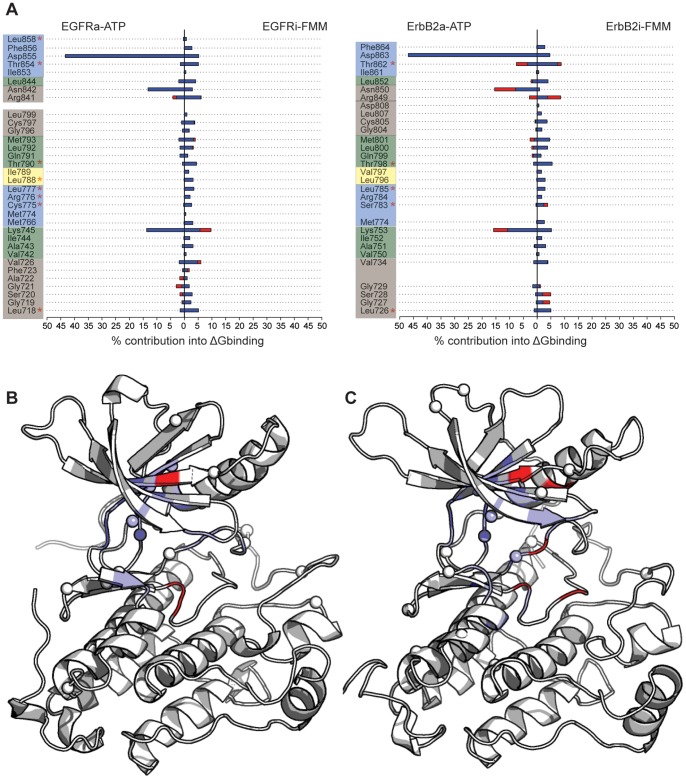
*In silico* predicted resistance mutations. The normalized values of the residuewise contribution to the binding energy are shown for EGFR and ErbB2 (A). Those residues to the left of the 0 line are involved in the binding of ATP, while the ones to the right contribute to the binding of lapatinib. The colored boxes follows the same color scheme than in Figure 4, with the exception of the hinge region that here is combined with the regions that make up the adenine pocket. The position of those residues on the three-dimensional structures of EGFR (B) and ErbB2 (C) have been mapped. The residues important in the binding of ATP are colored in red, while the ones involved in the binding of lapatinib are colored in blue. For reference, the experimental-based resistance mutations are shown in red stars in (A), and with spheres in (B) and (C).

Lapatinib selectively binds to the inactive conformation of the kinase domain. Targeting the inactive conformation results in higher selectivity compared with TKIs directed against the more conserved active state. However, the stringent conformational requirements for the binding render lapatinib a strong candidate to raise resistance, although it has not yet been reported in clinics. To examine what residues are chiefly responsible for the binding of lapatinib relative to ATP, the different residue-wise contributions to the total binding energy extracted from alanine scanning for the active and inactive kinases bound to ATP and to lapatinib have been calculated for the tighter and looser binding subpopulations. The interaction profiles are shown in Figure 7A for EGFR and ErbB2 and the positions of these residues are mapped on to the three dimensional structures of the kinases (Figure 7B and 7C for EGFR and ErbB2 respectively). For comparison purposes and as a benchmark to validate our approach, the resistant mutations found in laboratory-based experiments (Figure 1) have been marked in the profile plots and as spheres in the cartoon figures.

It is clear and not surprising that most of the predicted sites for putative lapatinib-resistant mutations cluster in and around specific regions surrounding the binding site. Some of the residues identified in our study overlap with those reported in the experimental setting. In the deep selectivity pocket at the back of the ATP binding cleft, where the m-flourobenzyloxy group sits, Cys775, Met776, Leu777, Leu788 for ErbB2 and Ser783, and Leu785 in EGFR directly contact lapatinib; mutations that destabilize the hydrophobic interactions with the aniline group will induce an escape mechanism. The P-loop region shows a large contribution to the binding of lapatinib, so mutations, in particular at Leu718/Leu726 (EGFR/ErbB2) will likely reduce the potency of the inhibitor. In the case of the binding of ATP, although these residues participate in the binding, their contributions are significantly smaller. The activation loop, and in particular the preceding residues interact with lapatinib;Thr854/Thr862 in EGFR/ErbB2 have been found to mutate in laboratory settings, leading to resistance against the inhibitor.

Apart from the amino acids near the ATP-binding pocket whose mutations have the potential to attenuate lapatinib binding, there are some residues that are not within contact-distance to lapatinib and the alternative mechanisms by which these mutations confer resistance are more diverse and have been hypothesized to modulate the dynamics or alter the equilibrium between the different conformations the kinase adopts. Two stretches of high importance are the residues preceding the αC-helix (Thr845 or Arg899 for EGFR) and the activation loop (such as Arg875 and His787 in EGFR), since they might destabilize the closed conformation in favor of the active state, thereby counteracting lapatinib inhibition. These may emerge either in much longer simulations or may result from more complex mechanisms than can be accounted for by the models currently being used.

The approach we have taken here reveals residues that appear to be critical for association of lapatinib with EGFR/ErbB2 and are likely sites for the emergence of resistant mutations that will render lapatinib ineffective; clinical data is eagerly awaited. Some of the residues are located in the two β-sheets connected by the P-loop, with special relevance to Valine at position 726 in EGFR and 734 for ErbB2. Paradoxically the Thr733 has been experimentally detected to be a spot for resistance. The three residues preceding the catalytic lysine, and a long stretch after the gatekeeper threonine significantly contribute in the binding for both kinases, and mutations at these sites will lead to changes in the morphology of the bottom of the pocket and thus the binding of inhibitor. We hypothesize that these residues are potential fragile points that can be mutated for the kinases to acquire resistance.

## Discussion

Drug-imposed selection processes inevitably will give rise to resistance against targeted therapies with one route to resistance arising from mutations in the target proteins. Among the kinases, these inhibitor desensitizing mutations will be constrained by the need to retain both, the binding of the substrate ATP, and the incumbent catalytic activity. Protein structural changes associated with specific mutations have been recognized as important information for the design of cancer therapeutics [23], however structure-based drug design has rarely exploited such information. Accordingly, mechanistic and structural insights into the molecular aspects of drug-target resistance can provide a rationale for the design and development of a set of distinct small molecules inhibitors with potent activity selective against desensitized kinase mutant alleles and improved resistance profiles.

In order to achieve a deeper understanding, it is essential to characterize the conformational landscape of these kinases and their functional mutant forms to provide access to unique or hidden conformations that are rarely exploited in biochemical and structural experiments, where typically only the active state of the enzyme is used.

In the current study, our aim was to investigate structural, dynamical and energetics effects in these associations, in an attempt to characterize different binding modes that can be targeted by new and more specific inhibitors. Indeed Rizzo’s group has introduced the concept of variability in the structures and ensemble of populations, by modeling different initial structures for their calculations [21]. Even though they concluded that a global similarity in the residue-wise interactions appears to exist, they have identified residues showing different contributions in the binding for the different models. In our model, we have used a recently described protocol [22] based on describing subpopulations that encompass the full binding-energy landscape from multiple long simulations. The approach we have followed in this study presents certain advantages that we summarize here:

application of improved statistical methods for the detection of different subpopulations within the bound state of these proteins. This guarantees a more efficient detection of residues that might be involved in the binding either in a transient conformation or even in a long lived state that has not been characterized earlier. These different binding modes extracted from an exhaustive search in the conformational space can be incorporated into the construction of pharmacophores with an expanded chemical space.
water molecules seem to play a critical role in the binding of small molecules for this system [21]. We have included for both inhibitor and natural ligand the effect of some key water molecules by explicitly including them in our input model.

The predictive ability of our approach yields a list of residues with a high tendency to be mutated and alter the binding of lapatinib. They correspond to residues that contribute significantly in the binding of lapatinib but participate minimally in the association of ATP (Figure 7A). While realistic quantitative predictions of these contributions is difficult currently owing to deficiencies in force fields and lack of exhaustive sampling [24], our approach begins to distill out the differing contributions within the different subpopulations thus highlighting the importance of accounting for this significant heterogeneity of conformations. The putative mutants appear to be confined to regions adjacent to the binding pockets of the enzymes (Figure 3 and Figure 4). Lapatinib buries its m-flourobenzyloxy moiety deep in the selectivity pocket making extensive contacts, which have been already reported to confer resistance (see box blue in Figure 4B). Important and novel hotspots are also found in the hydrophobic pocket I, such as those adjacent to the P-loop hairpin (Ser720/728, Val726/734), and the stretch after the hinge region (Cys797/805). Finally it is worth mentioning the residues in the hinge region together with those defining the adenine pocket that mainly contact the quinazoline ring of lapatinib. This region is delimited by residues contiguous to but following the gatekeeper threonine and those preceding the catalytic lysine.

We are currently extending this study to the incorporation of more complex models to complement the exhaustive study presented here. The availability of data from the clinic in response to lapatinib therapy will test the validity of such studies and undoubtedly inform us, helping in the improvement of the models employed towards the design of better inhibitors that can combat resistance.

## Methods

### Initial Preparation of the Models

The coordinates for the different systems shown in Table S1 were obtained from previous work in our lab [25]. For the active conformations, in addition to an ATP molecule, two Mg2+ ions and three catalytically important water molecules (here called key waters) were retained in the initial structures. The constructs for the tyrosine kinase domains consist of residues 693 to 991 and 701 to 999 for EGFR and ErbB2 respectively (the numbering throughout this article includes the 24-residue signal sequence). Standard topology and parameters files were used for ATP and Mg2+. The restrained electrostatic potential (RESP) method was used to generate the partial atomic charges of lapatinib, while the general AMBER force field GAFF [26] was used to assign the parameters. For the protein parameters we have used the AMBER ff99SB all-atom force field [27], together with the tleap module of AMBER for the solvation and neutralization of the systems. Each structure was explicitly solvated using the TIP3P model for water [28] and with the buffering distance set to a minimum of 10 Å. Sodium (Na+) and chloride (Cl−) ions were added to achieve net electroneutrality of the system.

### Simulations

Minimization and MD simulations were carried out using the Sander module of the Amber12 package [29] using the GPU-accelerated version of the program [30]. Periodic boundary conditions were applied, with van der Waals interactions trunctated at 8 Å and the particle mesh Ewald algorithm [31] adopted for the calculation of long-range electrostatic interactions. The integration time step was set to 2.0 fs and the SHAKE algorithm was employed to constrain the lengths of all chemical bonds involving hydrogen atoms at their equilibrium values. We have followed a protocol with the following steps: a) an initial minimization protocol involving 2000 steps of minimization with steepest descent followed by 1000 of conjugate gradient and with initial restraints on the solute, followed by restraints on the water and finally an unrestrainted minimization. After this minimization protocol, we will slowly heat the system up to room temperature (300 K) using six 50 ps-heating stages with 0.5 fs time step. This was followed by 50 ps of density equilibration with weak restraints on the system and finally 2 ns of constant pressure equilibration at 300 K with Langevin dynamics as temperature controller [ref]. After equilibration, production runs of 50 ns were performed for each system. In order to provide enough sampling of the system, which it is required for the segmentation protocol followed here (see next section), three replicates of 50 ns were obtained for every system included in this work.

#### Detection of relevant water molecules: hydrogen bond analysis

The hydrogen bonding analysis, including those involving the explicit water molecules, for the different systems was carried out using the cpptaj and ptraj modules within Amber suite of programs using standard criteria. We have also calculated the B-factors for all the explicit water molecules in the system, and those with a value comparable to the atoms in the protein were kept, under the assumption that the water molecules at these positions do not exchange with the bulk solvent due to important interactions with the system (protein and ligand). In the case of the active kinases, we have kept three water molecules while for the inactive states, only one water molecule has been found to be in a key position bridging the lapatinib and the kinase.

### Estimation of Binding Energies

The MMPBSA.py script in AMBER 11 suite was used to obtain estimates of the changes in binding free energy based on the trajectories. The molecular mechanical energies were calculated with the sander module; the polar contribution was calculated using the modified generalized Born model described by Onufriev et al [32]. The solvent accessible surface area was obtained with the molsurf program [33].

### Identification of Co-existing Populations: Binding Energy Segmentation

The simulations presented in this study are longer than those normally carried out [34], [35]. The idea behind extending the simulations to 50 ns is to enhance the sampling of the conformational space with a view to capturing alternate modes of interactions between the ligands and the proteins. Towards this, we apply our recently developed approach, the MMPBSA_segmentation [22] to distinguish different populations in the binding modes of ATP and lapatinib to EGFR and ErbB2 using three replicates of 50 ns. These discrete states and their descriptors are summarized in Table 1 and graphically represented in Figure 2A–D.

### Characterization of the Different Subpopulations: Structural and Dynamical Analysis and Alanine Scanning

Once the different populations were identified, computational alanine scanning was performed using the alanine scanning module implemented in MM-PBSA method of Amber 12. This protocol was carried out as follows: (1) for every subpopulation, we merged the different stretches identified as described above and extracted snapshots every 10 ps. (2) For each snapshot (after removing all waters), we mutated each residue in the the kinases to alanine. (3) For each snapshot, we obtained the binding free energy ΔG for both the wild type (ΔG_wt_) and each alanine mutant (ΔG_mut_). The difference in ΔG (ΔΔG = ΔG_mut_−ΔG_wt_) is used to estimate the contribution of each residue in the binding of both ATP and lapatinib.

The ptraj and cpptraj modules of AMBER 12 have been used for the analysis carried out in this study. PyMOL [36] and Visual Molecular Dynamics (VMD) [37] were used to visualize and generate the figures. R package has been used for generating the graphs.

## Supporting Information

Figure S1 **Conformational sampling for the different systems** during three independent 50 ns MD simulations. The root-mean square deviation (RMSD) of the Cα atoms of the systems studied here with respect to first frame in each trajectory as a function of time is shown for EGFR (A) and ErbB2 (B) bound to the natural ligand, and EGFR (C) and ErbB2 (D) bound to the inhibitor lapatinib.
(TIF)

Figure S2 **Density plots of the RMSD versus the total binding energies** for EGFR (A) and ErbB2 (B) bound to ATP, and EGFR (C) and ErbB2 (D) bound to lapatinib.
(TIF)

Figure S3 **Molecular interactions in the associations of EGFR and ErbB2.** (A) Hydrogen bond interactions found in the active EGFR in association with the natural ligand ATP; (B) Hydrogen bonds detected in the inactiva ErbB2 kinase when bound to lapatinib.
(TIF)

Figure S4 **Secondary structure evolution.** The secondary structure of every system has been represented as a function of time for EGFR (A) and ErbB2 (B) bound to ATP, and EGFR (C) and ErbB2 (D) bound to lapatinib. Helices are colored in blue, sheets in red, turns in black, 3–10 helix in green and pi helix in cyan, while absence of secondary structure is colored in yellow.
(TIF)

Table S1 **Different systems for which MD simulations have been carried out in this study.**
(DOC)

Table S2 **Hydrogen bond interactions in EGFRa bound to ATP.2MG.3HOH.**
(DOC)

Table S3 **Hydrogen bond interactions in ErbB2a bound to ATP.2MG.3HOH.**
(DOC)

Table S4 **Mg2+ coordination sphere in EGFRa bound to ATP.2MG.3HOH.**
(DOC)

Table S5 **Mg2+ coordination sphere in ErbB2a bound to ATP.2MG.3HOH.**
(DOC)

Table S6 **Hydrogen bond interactions in EGFRi bound to FMM.1HOH.**
(DOC)

Table S7 **Hydrogen bond interactions in ErbB2i bound to FMM.1HOH.**
(DOC)

Table S8 **van der Waals interactions in EGFRa bound to ATP.2MG.3HOH.**
(DOC)

Table S9 **van der Waals interactions in ErbB2a bound to ATP.2MG.3HOH.**
(DOC)

Table S10 **van der Waals interactions in EGFRi bound to FMM.1HOH.**
(DOC)

Table S11 **van der Waals interactions in ErbB2i bound to FMM.1HOH.**
(DOC)
