# Supplementary figures and images for: Differences in the Binding Affinities of ErbB Family: Heterogeneity in the Prediction of Resistance Mutants
Source: PLoS One. 2013 Oct 23;8(10):e77054. doi: 10.1371/journal.pone.0077054 (PMC3806757; doi:10.1371/journal.pone.0077054)

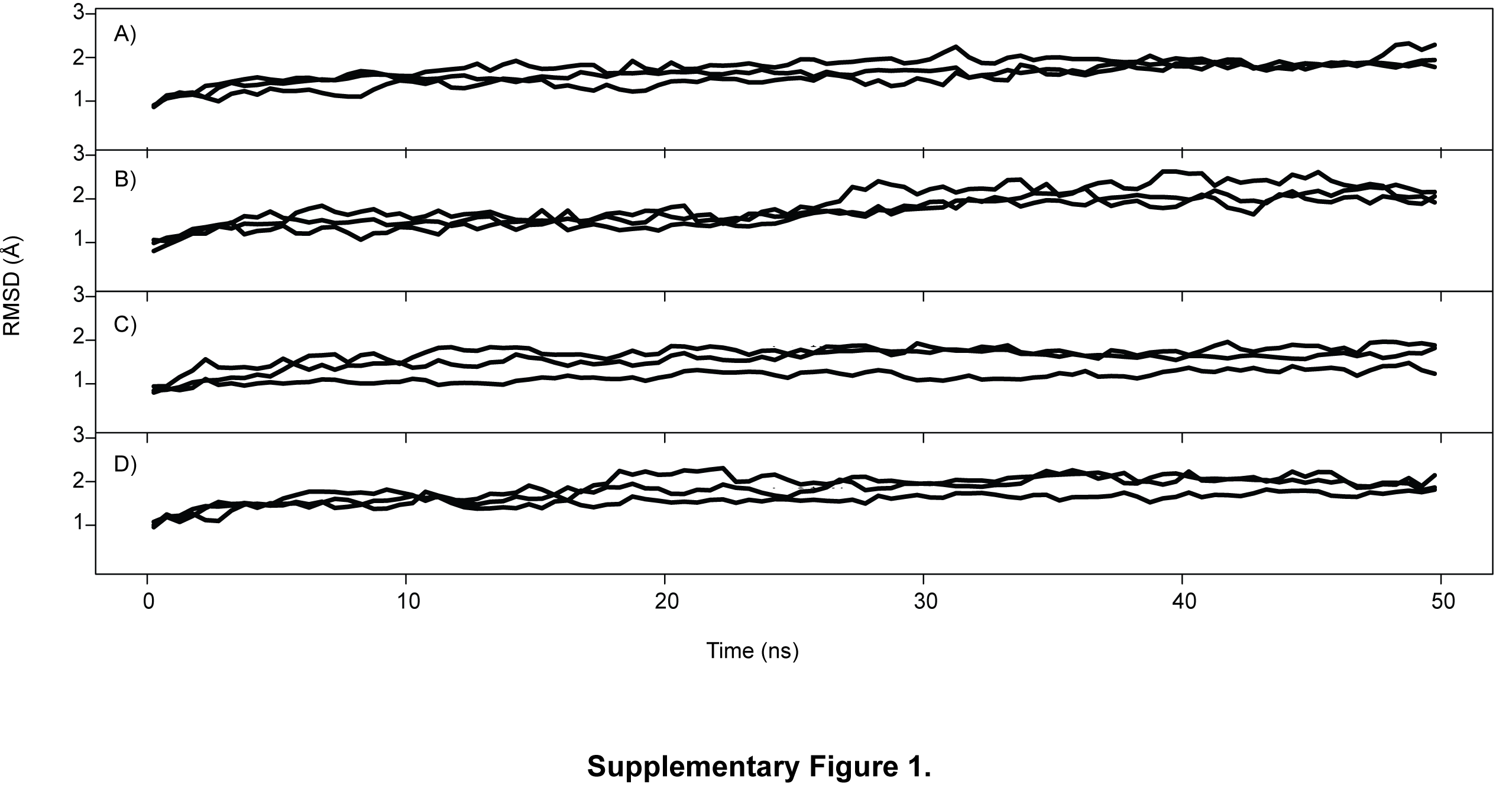

Supplement: Figure S1 — Conformational sampling for the different systems during three independent 50 ns MD simulations. The root-mean square deviation (RMSD) of the Cα atoms of the systems studied here with respect to first frame in each trajectory as a function of time is shown for EGFR (A) and ErbB2 (B) bound to the natural ligand, and EGFR (C) and ErbB2 (D) bound to the inhibitor lapatinib. (TIF) [file pone.0077054.s001.tif]

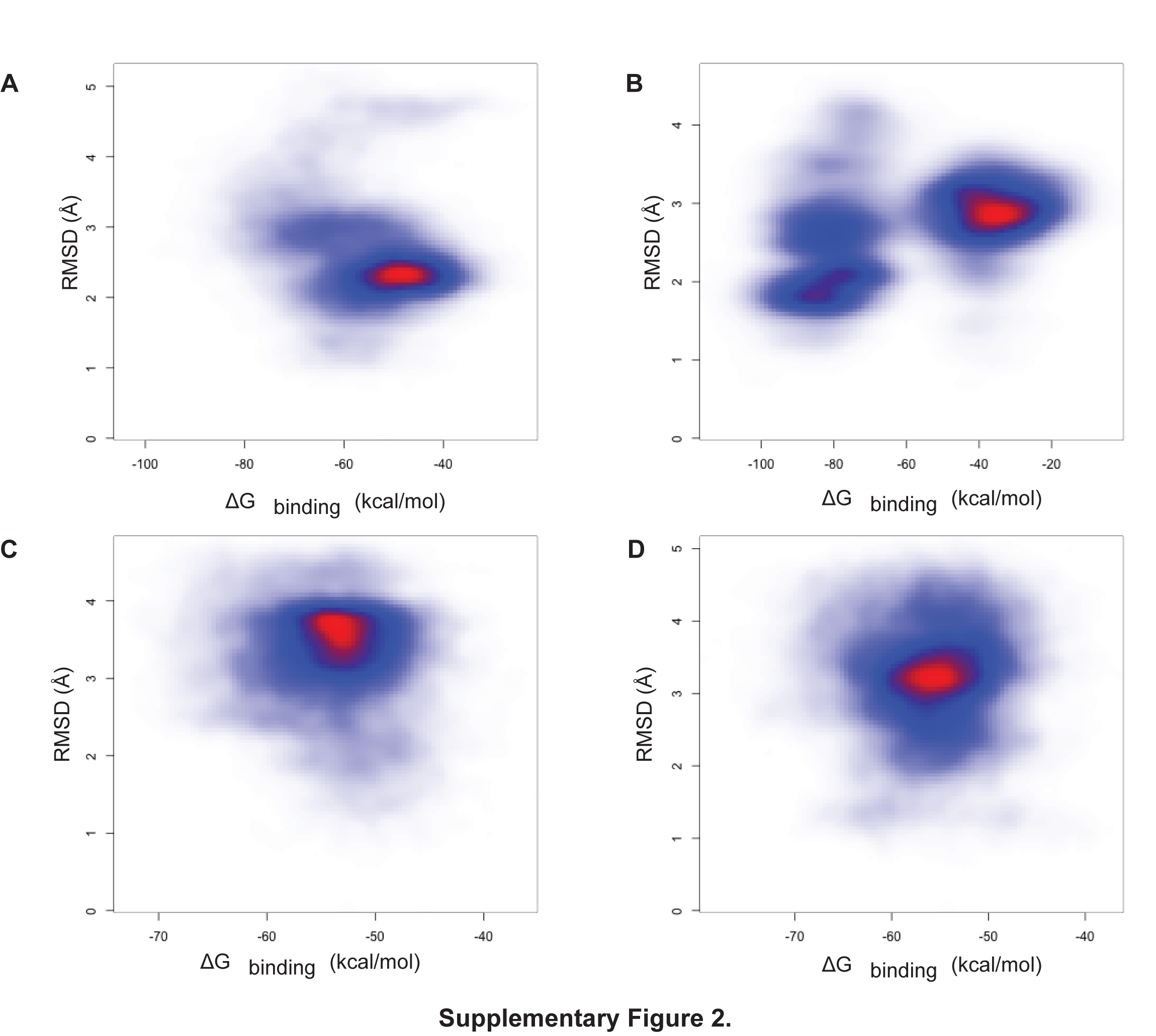

Supplement: Figure S2 — Density plots of the RMSD versus the total binding energies for EGFR (A) and ErbB2 (B) bound to ATP, and EGFR (C) and ErbB2 (D) bound to lapatinib. (TIF) [file pone.0077054.s002.tif]

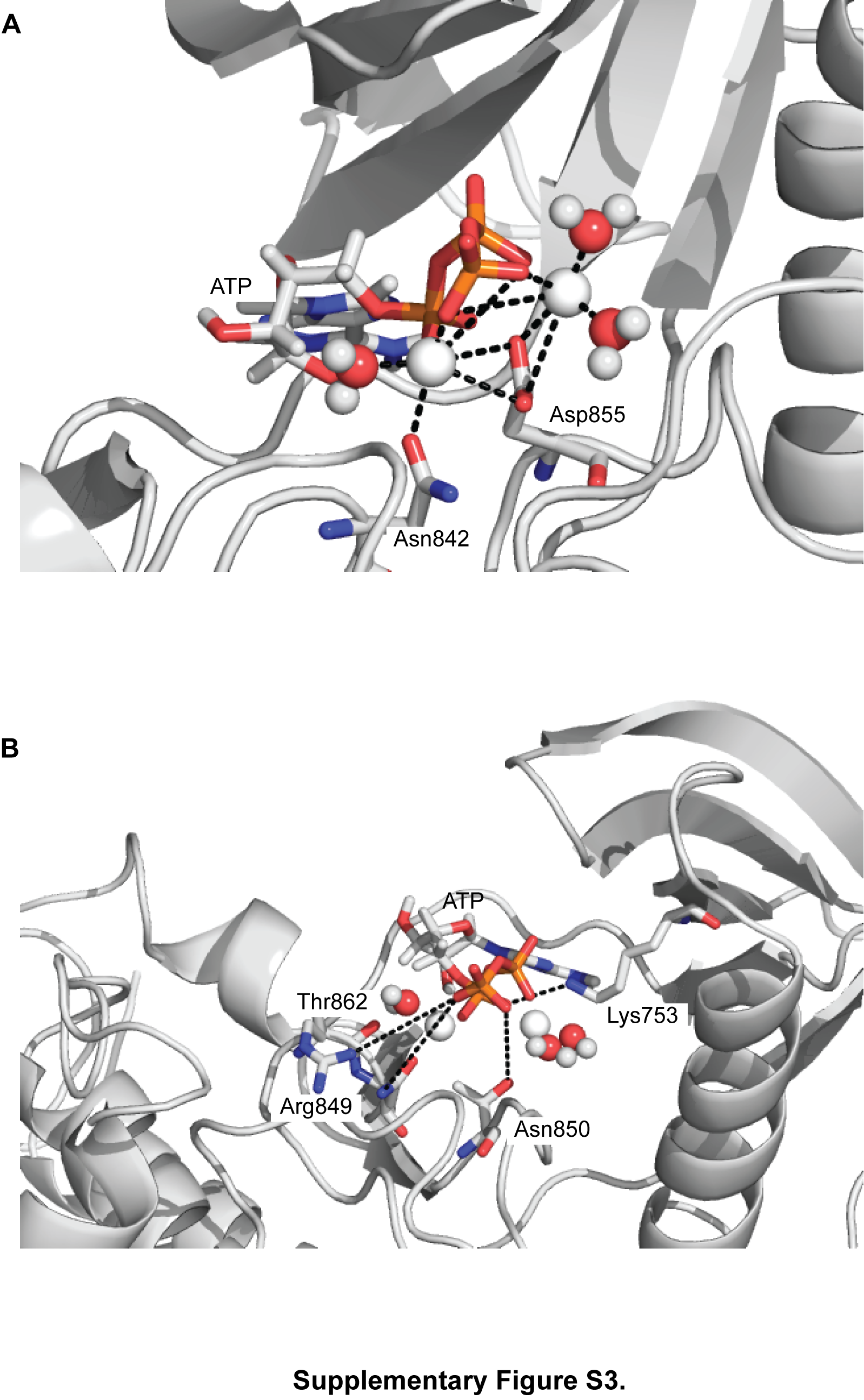

Supplement: Figure S3 — Molecular interactions in the associations of EGFR and ErbB2. (A) Hydrogen bond interactions found in the active EGFR in association with the natural ligand ATP; (B) Hydrogen bonds detected in the inactiva ErbB2 kinase when bound to lapatinib. (TIF) [file pone.0077054.s003.tif]

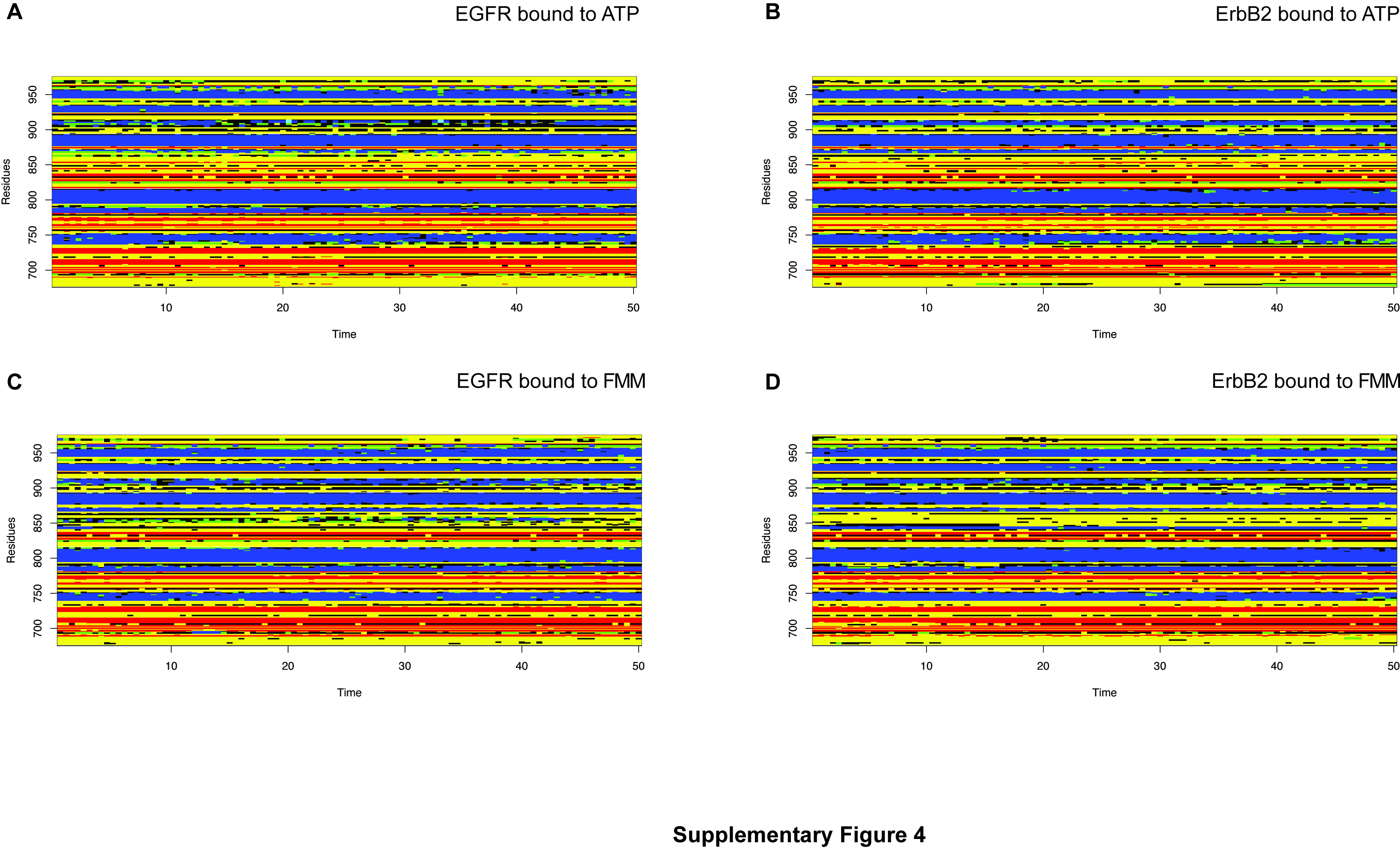

Supplement: Figure S4 — Secondary structure evolution. The secondary structure of every system has been represented as a function of time for EGFR (A) and ErbB2 (B) bound to ATP, and EGFR (C) and ErbB2 (D) bound to lapatinib. Helices are colored in blue, sheets in red, turns in black, 3–10 helix in green and pi helix in cyan, while absence of secondary structure is colored in yellow. (TIF) [file pone.0077054.s004.tif]
