# Supplementary material for: Differences in the Binding Affinities of ErbB Family: Heterogeneity in the Prediction of Resistance Mutants
Source: PLoS One. 2013 Oct 23;8(10):e77054. doi: 10.1371/journal.pone.0077054 (PMC3806757; doi:10.1371/journal.pone.0077054)
Supplement: Table S1 — Different systems for which MD simulations have been carried out in this study. (DOC) [file pone.0077054.s005.doc]

**Table S1**. Different systems for which MD simulations have been carried out in this study.

| System* | Kinase | State | Ligand |
| --- | --- | --- | --- |
| EGFRa-ATP | EGFR | active | ATP.2MG.3WAT |
| EGFRi-FMM | EGFR | inactive | lapatinib.1WAT |
| ErbB2a-ATP | ErbB2 | active | ATP2Mg3WAT |
| ErbB2i-FMM | ErbB2 | inactive | lapatinib |

* the letter a or i after the kinase name indicate the kinase state, i.e.: if the kinase is active or inactive, respectively.

** for the ligand ATP, 2 MG2+ ions and 3 water molecules were considered as ligand in order to provide stabilization. For the lapatinib, 1 water molecule was included.

Information concerning hydrogen bonds, ionic interactions with magnesium ions and van der Waals contacts for the different systems analyzed in this study. The values shown in these tables correspond to the % occupation, which it means the percentage of interaction populated over the whole MD trajectory. This observable evaluates the stability and the strength of such an interaction. Only those pair of atoms with a higher value than 20 are reported in these tables.

All the groups identified upon the segmentation protocol are shown here. The tighter binding mode has been colored in red, while the looser binder is highlighted in blue.
