# Supplementary material for: Differences in the Binding Affinities of ErbB Family: Heterogeneity in the Prediction of Resistance Mutants
Source: PLoS One. 2013 Oct 23;8(10):e77054. doi: 10.1371/journal.pone.0077054 (PMC3806757; doi:10.1371/journal.pone.0077054)
Supplement: Table S2 — Hydrogen bond interactions in EGFRa bound to ATP.2MG.3HOH. (DOC) [file pone.0077054.s006.doc]

**Table S2**. Hydrogen bond interactions in EGFRa bound to ATP.2MG.3HOH.

|  | **grp1** | **grp2** | **grp3** | **grp4** |
| --- | --- | --- | --- | --- |
|  |  |  |  |  |
| Lys745@NZ--WAT2@O | 43 | 43 | 43 |  |
| Lys745@NZ--ATP@O3A | 37 | 37 |  | 38 |
| Lys745@NZ--ATP@O1B | 96 | 98 | 97 | 97 |
| Lys745@NZ--ATP@O1A | 99 | 99 | 61 | 100 |
| Glu762@OE1-WAT1@O |  |  | 57 |  |
| Glu762@OE1-WAT2@O | 66 | 93 | 92 |  |
| Thr790@OG1-ATP@N6 |  |  |  | 46 |
| Gln791@O-ATP@N6 | 58 | 90 | 99 | 99 |
| Met793@O-ATP@N6 | 37 |  |  |  |
| Arg841@O-WAT3@O | 83 | 88 | 99 | 97 |
| Arg841@NH2--ATP@O1G | 26 | 26 |  |  |
| Arg841@NH1--ATP@O2G | 30 | 40 | 27 |  |
| Arg841@NH1--ATP@O1G | 31 | 51 | 27 |  |
| Arg841@NH1-ATP@OG1 | 31 |  |  |  |
| Asn842@OD1-WAT3@O | 65 | 65 | 66 | 67 |
| Asp855@OD1-WAT1@O | 70 | 71 | 98 | 62 |
| Asp855@OD1-WAT2@O | 56 | 56 | 89 |  |
| Asp855@OD2-WAT3@O | 32 |  | 97 |  |
