# Supplementary material for: Differences in the Binding Affinities of ErbB Family: Heterogeneity in the Prediction of Resistance Mutants
Source: PLoS One. 2013 Oct 23;8(10):e77054. doi: 10.1371/journal.pone.0077054 (PMC3806757; doi:10.1371/journal.pone.0077054)
Supplement: Table S3 — Hydrogen bond interactions in ErbB2a bound to ATP.2MG.3HOH. (DOC) [file pone.0077054.s007.doc]

**Table S3.** Hydrogen bond interactions in ErbB2a bound to ATP.2MG.3HOH.

|  | **grp1** | **grp2** | **grp3** | **grp4** | **grp5** |
| --- | --- | --- | --- | --- | --- |
| Lys753@NZ--**ATP@O1A** |  |  |  | 77 | 40 |
| Lys753@NZ--ATP@O2B | 81 | 53 | 98 | 66 | 88 |
| Lys753@NZ--ATP@O1B |  | 34 |  | 81 | 42 |
| Lys753@NZ--ATP@O3A | 39 |  | 47 | 58 | 68 |
| Lys753@NZ-ATP@O5’ | 64 |  | 93 |  |  |
| Glu770@OE1-WAT1@O |  | 61 |  |  |  |
| Glu770@OE1-WAT2@O |  | 38 |  | 38 |  |
| Glu770@OE2-WAT1@O |  | 46 |  | 85 | 42 |
| Glu770@OE2-WAT2@O |  | 70 |  | 70 | 42 |
| Thr798@OG1-ATP@N6 |  | 38 |  | 76 | 86 |
| Gln799@O-ATP@N6 | 99 | 99 | 100 | 99 | 97 |
| Asp845@OD1-WAT1@O |  |  | 89 |  |  |
| Asp845@OD2-WAT1@O | 81 |  |  |  | 55 |
| Arg849@NH1-ATP@OG1 |  |  | 87 | 31 |  |
| Arg849@NH1--ATP@O2G |  |  | 87 | 31 |  |
| Arg849@NH1--ATP@O3G |  |  | 91 | 46 |  |
| Arg849@NH2--ATP@O1G |  |  | 25 | 35 |  |
| Arg849@NH2--ATP@O3G |  |  | 96 |  |  |
| Arg849@NH2--WAT1@O |  |  | 89 |  |  |
| Arg849@O-WAT3@O | 76 |  | 84 |  | 74 |
| Asn850@ND2-ATP@OG2 | 37 |  | 48 |  |  |
| Asn850@ND2-ATP@O3G | 78 |  | 84 |  |  |
| Asn850@OD1--WAT@O | 70 | 90 | 71 | 58 | 53 |
| Thr862@OG1--ATP@O1A | 81 |  | 98 | 24 | **54** |
| Asp863@OD1-WAT1@O | 57 |  | 76 |  | 56 |
| Asp863@OD1-WAT2@O | 62 |  | 77 |  |  |
| Asp863@OD1-WAT3@O |  |  | 67 |  | 40 |
| Asp863@OD2-WAT1@O |  | 78 | 67 |  | 66 |
| Asp863@OD2-WAT2@O | 95 | 92 | 100 |  | 56 |
