# Supplementary material for: Differences in the Binding Affinities of ErbB Family: Heterogeneity in the Prediction of Resistance Mutants
Source: PLoS One. 2013 Oct 23;8(10):e77054. doi: 10.1371/journal.pone.0077054 (PMC3806757; doi:10.1371/journal.pone.0077054)
Supplement: Table S4 — Mg2+ coordination sphere in EGFRa bound to ATP.2MG.3HOH. (DOC) [file pone.0077054.s008.doc]

**Table S4.** Mg2+ coordination sphere in EGFRa bound to ATP.2MG.3HOH.

|  | **grp1** | **grp2** | **grp3** | **grp4** |
| --- | --- | --- | --- | --- |
| Glu762@OE2--MG1 | 29 | 22 | 51 |  |
| Glu762@OE1--MG1 | 22 | 26 | 57 |  |
| Asp855@OD2--MG1 | 100 | 100 | 100 | 100 |
| Asp855@OD1--MG2 | 100 | 100 | 100 | 100 |
| Asp855@OD1--MG1 | 100 | 100 | 100 | 100 |
| MG1--WAT2@O | 100 | 100 | 100 | 100 |
| MG1--WAT1@O | 100 | 100 | 100 | 100 |
| MG1--ATP@O3G | 100 | 100 | 100 | 100 |
| MG1--ATP@O2G | 100 | 100 | 100 | 100 |
| MG1--ATP@O1G | 59 | 63 | 26 | 57 |
| Glu837@OD2--MG2 |  | 33 |  | 21 |
| Glu837@OD1--MG2 | 23 |  |  |  |
| Asn842@OD1--MG2 | 100 | 100 | 100 | 100 |
| Asp855@OD2--MG2 | 100 | 100 | 100 | 100 |
| MG2-WAT3@O | 100 | 100 | 100 | 100 |
| MG2--ATP@O3G | 100 | 100 | 100 | 100 |
| MG2--ATP@O2G | 100 | 100 | 100 | 100 |
| MG2--ATP@O1G | 27 | 32 | 38 | 31 |
