# Supplementary material for: Differences in the Binding Affinities of ErbB Family: Heterogeneity in the Prediction of Resistance Mutants
Source: PLoS One. 2013 Oct 23;8(10):e77054. doi: 10.1371/journal.pone.0077054 (PMC3806757; doi:10.1371/journal.pone.0077054)
Supplement: Table S5 — Mg2+ coordination sphere in ErbB2a bound to ATP.2MG.3HOH. (DOC) [file pone.0077054.s009.doc]

**Table S5.** Mg2+ coordination sphere in ErbB2a bound to ATP.2MG.3HOH.

|  | **grp1** | **grp2** | **grp3** | **grp4** | **grp5** |
| --- | --- | --- | --- | --- | --- |
| MG1--ATP@O1G | 92 | 52 | 100 | 57 | 33 |
| MG1--ATP@O2G | 67 | 99 | 64 | 94 | 98 |
| MG1--ATP@O3G | 100 | 100 | 100 | 100 | 100 |
| MG1--WAT1@O | 100 | 100 | 100 | 100 | 100 |
| MG1--WAT2@O | 100 | 100 | 100 | 100 | 100 |
| Glu770@OE1--MG1 |  | 23 |  | 88 | 42 |
| Glu770@OE2--MG1 |  | 34 |  | 88 | 43 |
| MG1--Asp845@OD1 |  |  | 43 |  |  |
| MG1--Asp845@OD2 | 53 |  |  |  | 35 |
| MG1--Asp863@OD1 | 100 | 100 | 100 | 100 | 100 |
| MG1--Asp863@OD2 | 100 | 100 | 100 | 100 | 100 |
| MG2--ATP@O1G | 38 | 35 | 59 | 59 | 27 |
| MG2--ATP@O2G | 100 | 100 | 100 | 100 | 100 |
| MG2--ATP@O3G | 99 | 100 | 96 | 100 | 100 |
| MG2--WAT1@O |  |  |  | 38 | 38 |
| MG2--WAT3@O | 100 | 100 | 100 | 100 | 100 |
| Asp845@OD2--MG2 |  |  |  | 22 | 33 |
| Asn850@OD1--MG2 | 100 | 100 | 100 | 73 | 84 |
| Asp863@OD1--MG2 |  | 87 |  | 100 | 100 |
| Asp863@OD2--MG2 |  | 86 |  | 100 | 99 |
