# Supplementary material for: Differences in the Binding Affinities of ErbB Family: Heterogeneity in the Prediction of Resistance Mutants
Source: PLoS One. 2013 Oct 23;8(10):e77054. doi: 10.1371/journal.pone.0077054 (PMC3806757; doi:10.1371/journal.pone.0077054)
Supplement: Table S6 — Hydrogen bond interactions in EGFRi bound to FMM.1HOH. (DOC) [file pone.0077054.s010.doc]

**Table S6**. Hydrogen bond interactions in EGFRi bound to FMM.1HOH.

|  | **grp1** | **grp2** | **grp3** | **grp4** | **grp5** |
| --- | --- | --- | --- | --- | --- |
| Leu718@O--FMM@N7 | 32 |  |  |  |  |
| Ser720@O--FMM@N7 | 42 | 22 |  |  |  |
| Gly721@N--FMM@O4 |  |  |  | 24 |  |
| Ala722@N--FMM@O4 |  | 28 |  |  |  |
| Thr725@N--FMM@O4 |  |  |  |  | 36 |
| Val726@N--FMM@O4 |  |  |  |  | 66 |
| Lys745@NZ--FMM@O3 |  |  |  |  | 52 |
| Thr790@OG1--FMM@N20 | 45 | 26 | 90 | 81 | 72 |
| Met793@N--FMM@N18 | 53 | 30 | 95 | 87 | 80 |
| Asp800@N--FMM@O3 |  |  |  | 23 |  |
| Thr854@OG1--WAT@O |  | 25 | 90 | 51 |  |
| WAT@O--FMM@N20 | 56 | 71 | 21 | 52 | 93 |
