# Supplementary material for: Differences in the Binding Affinities of ErbB Family: Heterogeneity in the Prediction of Resistance Mutants
Source: PLoS One. 2013 Oct 23;8(10):e77054. doi: 10.1371/journal.pone.0077054 (PMC3806757; doi:10.1371/journal.pone.0077054)
Supplement: Table S7 — Hydrogen bond interactions in ErbB2i bound to FMM.1HOH. (DOC) [file pone.0077054.s011.doc]

**Table S7**. Hydrogen bond interactions in ErbB2i bound to FMM.1HOH.

|  | **grp1** | **grp2** | **grp3** | **grp4** |
| --- | --- | --- | --- | --- |
| Leu726@O--FMM@N7 |  | 35 | 27 |  |
| Ser728@N--FMM@N7 |  | 61 |  | 89 |
| Ser728@N--FMM@O4 |  | 29 |  |  |
| Ser728@OG--FMM@O4 |  |  |  | 46 |
| Ser783@OG--WAT@O |  |  | 22 | 44 |
| Thr798@OG1--FMM@N20 | 70 | 63 | 61 | 79 |
| Thr798@OG1--WAT@O | 32 |  | 70 | 70 |
| Met801@N--FMM@N18 | 81 | 83 | 83 | 87 |
| Arg811@NH1--FMM@O3 |  |  | 21 |  |
| Arg811@NH2--FMM@O3 |  |  | 21 |  |
| Arg849@NH1--FMM@O4 |  |  |  | 88 |
| Thr862@OG1--WAT@O | 50 | 43 | 99 | 98 |
