# Supplementary material for: Differences in the Binding Affinities of ErbB Family: Heterogeneity in the Prediction of Resistance Mutants
Source: PLoS One. 2013 Oct 23;8(10):e77054. doi: 10.1371/journal.pone.0077054 (PMC3806757; doi:10.1371/journal.pone.0077054)
Supplement: Table S8 — van der Waals interactions in EGFRa bound to ATP.2MG.3HOH. (DOC) [file pone.0077054.s012.doc]

**Table S8.** van der Waals interactions in EGFRa bound to ATP.2MG.3HOH.

|  | **grp1** | **grp2** | **grp3** | **grp4** |
| --- | --- | --- | --- | --- |
| Leu718@CD2--ATP@C2 |  | 60 | 54 | 53 |
| Leu718@CG--ATP@C2 | 77 | 90 | 79 |  |
| Leu718@CG--ATP@C4 | 73 | 65 |  |  |
| Ser720@C--ATP@C4' | 63 | 80 | 71 |  |
| Ser720@C--ATP@C5' | 67 | 84 | 86 |  |
| Gly721@CA--ATP@C5' |  | 61 | 63 |  |
| Gly721@CA--ATP@PG |  | 55 |  |  |
| Val726@CB--ATP@C8 |  |  | 51 |  |
| Val726@CG1--ATP@C1' |  |  | 69 |  |
| Val726@CG1--ATP@C4 |  |  | 55 |  |
| Val726@CG1--ATP@C5 |  | 54 |  |  |
| Val726@CG1--ATP@C8 |  |  |  | 62 |
| Val726@CG2--ATP@C4' |  | 51 |  |  |
| Val726@CG2--ATP@C5' | 60 | 60 |  |  |
| Val726@CG2--ATP@C8 |  | 51 |  |  |
| Ile744@CB--ATP@C6 |  | 62 |  | 88 |
| Ile744@CG2--ATP@C6 |  | 77 | 65 | 93 |
| Glu746@CA--ATP@PB |  |  |  | 64 |
| Glu746@CB--ATP@PA | 99 | 99 |  | 100 |
| Glu746@CB--ATP@PB | 96 | 98 |  | 96 |
| Glu746@CG1--ATP@PA | 65 | 64 |  | 66 |
| Glu746@CG1--ATP@PB | 70 | 70 |  | 68 |
| Pro794@CG--ATP@C2 | 81 | 91 | 100 | 98 |
| Pro794@CG--ATP@C6 | 81 | 89 | 94 | 61 |
| Leu798@CD1--ATP@C1' | 55 | 51 |  |  |
| Leu798@CD1--ATP@C2' | 81 | 79 |  | 65 |
| Leu798@CD1--ATP@C3' |  | 70 |  | 64 |
| Lys846@CD--ATP@C4 |  | 62 | 57 | 59 |
| Lys846@CD--ATP@C5 | 66 |  |  | 73 |
| Lys846@CD--ATP@C6 | 66 | 72 |  | 73 |
| Lys846@CE--ATP@C1' |  | 61 |  |  |
| Lys846@CE--ATP@C2 |  |  |  | 56 |
| Lys846@CE--ATP@C2' | 72 | 85 | 66 | 69 |
| Lys846@CE--ATP@C4 | 67 | 71 | 56 | 61 |
| Lys846@CE--ATP@C5 | 53 | 55 |  |  |
| Leu858@CD1--ATP@PA |  | 60 |  | 53 |
| Leu858@CD1--ATP@PB | 99 | 99 | 100 | 99 |
| Leu858@CD1--ATP@PG | 100 | 98 | 100 | 99 |
