# Supplementary material for: Differences in the Binding Affinities of ErbB Family: Heterogeneity in the Prediction of Resistance Mutants
Source: PLoS One. 2013 Oct 23;8(10):e77054. doi: 10.1371/journal.pone.0077054 (PMC3806757; doi:10.1371/journal.pone.0077054)
Supplement: Table S9 — van der Waals interactions in ErbB2a bound to ATP.2MG.3HOH. (DOC) [file pone.0077054.s013.doc]

**Table S9.** van der Waals interactions in ErbB2a bound to ATP.2MG.3HOH.

|  | **grp1** | **grp2** | **grp3** | **grp4** | **grp5** |
| --- | --- | --- | --- | --- | --- |
| Gly729@CA--ATP@PB |  |  |  | 58 |  |
| Leu726@CD2--ATP@C2 |  |  | 60 |  |  |
| Val734@CG2--ATP@C4' |  |  | 60 |  |  |
| Val734@CG2--ATP@C5' |  |  |  | 61 |  |
| Ala751@CB--ATP@C2 | 56 |  | 64 |  |  |
| Ala751@CB--ATP@C5 |  |  |  | 81 | 72 |
| Ala751@CB--ATP@C6 | 66 |  | 73 | 97 | 95 |
| Lys753@CE--ATP@C5' | 65 |  | 84 |  |  |
| Leu800@CD1--ATP@C2 | 71 | 58 | 74 | 60 | 70 |
| Met801@CB--ATP@C2 |  | 55 |  | 71 |  |
| Met801@CG--ATP@C6 |  |  | 52 |  |  |
| Cys805@CB--ATP@C3' |  | 65 |  |  |  |
| Arg849@CZ--ATP@PG |  |  | 94 |  |  |
| Leu852@CD1--ATP@C4 |  | 68 |  |  |  |
| Leu852@CD1--ATP@C5 | 81 | 63 | 97 | 68 | 68 |
| Leu852@CD1--ATP@C6 | 77 |  | 79 | 70 | 77 |
| Leu852@CD1--ATP@C8 |  |  | 54 |  |  |
| Leu852@CD2--ATP@C2 |  |  |  |  | 59 |
| Leu852@CD2--ATP@C2' |  |  |  | 62 | 54 |
| Leu852@CD2--ATP@C4 |  | 51 |  | 55 | 59 |
| Leu852@CD2--ATP@C5 |  | 58 |  |  |  |
| Thr862@CG2--ATP@PA | 65 |  | 78 |  |  |
| Asp863@CG--ATP@PA |  | 77 |  | 59 | 57 |
| Asp863@CG--ATP@PG |  | 79 |  |  |  |
