# Supplementary material for: Differences in the Binding Affinities of ErbB Family: Heterogeneity in the Prediction of Resistance Mutants
Source: PLoS One. 2013 Oct 23;8(10):e77054. doi: 10.1371/journal.pone.0077054 (PMC3806757; doi:10.1371/journal.pone.0077054)
Supplement: Table S10 — van der Waals interactions in EGFRi bound to FMM.1HOH. (DOC) [file pone.0077054.s014.doc]

**Table S10.** van der Waals interactions in EGFRi bound to FMM.1HOH.

|  | **grp1** | **grp2** | **grp3** | **grp4** | **grp5** |
| --- | --- | --- | --- | --- | --- |
| Leu718@C--FMM@C10 |  |  | 52 | 64 | 77 |
| Leu718@CB--FMM@C10 |  |  | 77 | 65 |  |
| Leu718@CB--FMM@C11 |  |  | 80 | 83 | 91 |
| Leu718@CB--FMM@C12 |  |  | 53 |  |  |
| Leu718@CD1--FMM@C11 |  |  | 57 |  |  |
| Leu718@CD1--FMM@C12 |  |  | 64 | 52 |  |
| Leu718@CD1--FMM@C14 |  |  | 82 | 71 |  |
| Leu718@CD1--FMM@C15 | 58 |  | 85 | 86 | 92 |
| Leu718@CD1--FMM@C16 |  |  |  |  | 64 |
| Gly719@CA--FMM@C8 |  |  |  | 57 |  |
| Ser720@C--FMM@C1 |  |  |  |  | 72 |
| Gly724ATP@CA--FMM@C1 |  |  |  |  | 52 |
| Gly724@C--FMM@C1 |  |  |  |  | 54 |
| Gly724@C--FMM@S2 |  |  |  |  | 75 |
| Val726@CG1--FMM@C39 |  | 56 |  |  | 52 |
| Val726@CG1--FMM@C40 | 51 | 71 | 56 | 61 | 93 |
| Val726@CG2--FMM@C5 |  |  |  |  | 74 |
| Val726@CG2--FMM@S2 |  |  |  |  | 59 |
| Ala743@CB--FMM@C17 |  |  | 91 | 81 | 66 |
| Ala743@CB--FMM@C19 | 98 | 98 | 98 | 98 | 100 |
| Ala743@CB--FMM@C21 | 53 |  | 90 | 81 | 65 |
| Ala743@CB--FMM@C39 |  |  | 80 | 65 |  |
| Ile744@C--FMM@C16 |  |  |  |  | 60 |
| Lys745@CB--FMM@C26 | 86 | 76 | 72 | 66 | 95 |
| Lys745@CB--FMM@C36 | 99 | 98 | 98 | 85 | 99 |
| Lys745@CB--FMM@C38 |  | 68 |  |  |  |
| Lys745@CB--FMM@Cl | 94 | 93 | 97 | 85 | 96 |
| Lys745@CD--FMM@C24 |  | 60 |  |  |  |
| Lys745@CD--FMM@C25 | 81 | 87 | 77 | 82 | 71 |
| Lys745@CD--FMM@C26 | 86 | 82 | 84 | 84 | 64 |
| Met766@CB--FMM@C30 |  | 58 |  |  |  |
| Met766@CB--FMM@C31 | 65 | 83 | 81 | 68 | 78 |
| Met766@CB--FMM@C32 |  |  | 53 |  |  |
| Met776@CG--FMM@C30 | 55 | 79 | 54 |  |  |
| Met766@CG--FMM@C31 | 81 | 81 | 83 | 76 | 83 |
| Cys775@C--FMM@F | 97 | 96 | 98 | 93 | 93 |
| Cys775@CA--FMM@F |  | 55 | 66 |  |  |
| Cys775@CB--FMM@F | 92 | 95 | 98 | 90 | 85 |
| Arg776@C--FMM@F | 100 | 99 | 99 | 98 | 98 |
| Arg776@CA--FMM@F | 91 | 96 | 95 | 89 | 80 |
| Leu777@CA--FMM@F | 76 | 70 | 59 | 77 | 80 |
| Leu777@CD1--FMM@C35 | 60 | 57 |  | 59 | 72 |
| Leu788@C--FMM@Cl | 100 | 99 | 100 | 100 | 100 |
| Leu788@CB--FMM@Cl | 51 | 69 | 84 | 64 | 56 |
| Leu788@CD1--FMM@C28 | 53 | 51 | 58 |  |  |
| Ile789@C--FMM@Cl | 88 | 80 | 88 | 94 | 98 |
| Ile789@CA--FMM@Cl | 83 | 73 | 82 | 97 | 98 |
| Thr790@CB--FMM@Cl | 73 | 74 | 51 | 60 | 67 |
| Thr790@CG2--FMM@C33 | 67 | 61 | 52 | 58 | 59 |
| Thr790@CG2--FMM@C35 | 93 | 94 | 90 | 90 | 91 |
| Thr790@CG2--FMM@C36 | 69 | 65 | 90 | 80 | 77 |
| Thr790@CG2--FMM@C38 |  |  | 51 |  |  |
| Thr790@CG2--FMM@Cl | 97 | 98 | 97 | 96 | 94 |
| Thr790@CG2--FMM@F | 98 | 96 | 96 | 95 | 96 |
| Leu792@CD1--FMM@C16 |  |  | 87 | 75 | 64 |
| Met793@CB--FMM@C16 |  |  | 51 |  |  |
| Met793@CG--FMM@C19 |  |  | 53 |  |  |
| Gly796@C--FMM@C15 |  |  | 59 |  |  |
| Gly796@CA--FMM@C11 |  |  | 57 |  |  |
| Gly796@CA--FMM@C15 | 55 |  | 80 | 86 | 92 |
| Gly796@CA--FMM@C16 | 55 |  |  | 61 | 76 |
| Cys797@CB--FMM@C10 |  |  |  |  | 53 |
| Cys797@CB--FMM@C11 |  |  |  |  | 53 |
| Cys797@CB--FMM@C12 |  |  |  |  | 77 |
| Cys797@CB--FMM@C15 |  | 61 |  |  |  |
| Cys797@CB--FMM@C9 |  |  |  |  | 70 |
| Arg84ATP@CD--FMM@C6 |  | 56 |  |  |  |
| Leu844@CD1--FMM@C17 |  |  | 67 | 58 | 58 |
| Leu844@CD1--FMM@C19 | 95 | 90 | 86 | 97 | 98 |
| Leu844@CD1--FMM@C21 | 64 |  | 96 | 98 | 97 |
| Leu844@CD1--FMM@C39 |  |  | 83 | 72 | 78 |
| Leu844@CD2--FMM@C17 | 74 | 68 |  | 57 | 57 |
| Leu844@CD2--FMM@C19 |  | 58 |  |  |  |
| Leu844@CD2--FMM@C21 | 54 |  |  |  | 62 |
| Leu844@CD2--FMM@C39 | 82 | 62 |  | 87 | 95 |
| Leu844@CD2--FMM@C40 |  |  | 51 | 79 | 93 |
| Thr854@CG2--FMM@C24 | 93 | 93 | 85 | 93 | 94 |
| Asp855@C--FMM@C30 | 54 | 79 | 81 | 61 |  |
| Asp855@C--FMM@C31 |  | 66 |  |  |  |
| Asp855@CA--FMM@C25 |  | 60 |  |  |  |
| Asp855@CA--FMM@C29 | 61 |  | 69 | 68 | 79 |
| Asp855@CA--FMM@C30 | 99 | 100 | 99 | 99 | 100 |
| Asp855@CA--FMM@C31 | 86 | 88 | 77 | 79 | 91 |
| Asp855@CB--FMM@C24 |  | 62 |  |  |  |
| Asp855@CB--FMM@C25 | 83 | 64 |  | 54 | 91 |
| Asp855@CG--FMM@C25 | 84 | 68 |  | 58 | 96 |
| Phe856@CD1--FMM@C31 |  |  | 53 |  |  |
| Phe856@CD2--FMM@C31 | 61 | 60 | 59 | 59 |  |
| Phe856@CE1--FMM@C31 | 59 | 56 | 67 | 57 |  |
| Phe856@CE2--FMM@C31 | 61 | 68 | 71 | 76 | 61 |
| Phe856@CE2--FMM@C32 | 91 | 91 | 87 | 87 | 83 |
| Phe856@CZ--FMM@C31 | 76 | 68 | 77 | 73 | 53 |
| Phe856@CZ--FMM@C32 | 87 | 92 | 95 | 88 | 73 |
