# Supplementary material for: Differences in the Binding Affinities of ErbB Family: Heterogeneity in the Prediction of Resistance Mutants
Source: PLoS One. 2013 Oct 23;8(10):e77054. doi: 10.1371/journal.pone.0077054 (PMC3806757; doi:10.1371/journal.pone.0077054)
Supplement: Table S11 — van der Waals interactions in ErbB2i bound to FMM.1HOH. (DOC) [file pone.0077054.s015.doc]

**Table S11.** van der Waals interactions in ErbB2i bound to FMM.1HOH.

|  | **grp1** | **grp2** | **grp3** | **grp4** |
| --- | --- | --- | --- | --- |
| Leu726@CD1--FMM@C14 | 57 |  |  |  |
| Leu726@CD1--FMM@C15 | 74 | 65 | 72 | 56 |
| Leu726@CD2--FMM@C14 |  |  |  | 71 |
| Leu726@CD2--FMM@C15 |  | 62 | 64 | 96 |
| Leu726@CD2--FMM@C16 |  | 51 | 66 | 93 |
| Leu726@CG--FMM@C14 |  |  | 52 | 76 |
| Leu726@CG--FMM@C15 |  |  | 65 | 95 |
| Gly727@CA--FMM@C10 |  |  |  | 82 |
| Gly727@CA--FMM@C8 |  |  |  | 71 |
| Gly727@CA--FMM@C9 |  |  | 67 | 99 |
| Val734@CG1--FMM@C40 |  |  |  | 70 |
| Ala751@CB--FMM@C17 | 73 | 60 | 66 | 85 |
| Ala751@CB--FMM@C19 | 99 | 99 | 100 | 100 |
| Ala751@CB--FMM@C21 | 86 | 64 | 81 | 88 |
| Ala751@CB--FMM@C39 | 56 |  |  | 57 |
| Ile752@C--FMM@Cl |  |  | 54 |  |
| Lys753@CB--FMM@C26 | 81 | 90 | 98 | 99 |
| Lys753@CB--FMM@C36 | 97 | 99 | 96 | 99 |
| Lys753@CB--FMM@Cl | 92 | 95 | 92 | 94 |
| Lys753@CD--FMM@C25 | 73 | 92 | 75 | 94 |
| Lys753@CD--FMM@C26 | 78 | 83 | 80 | 94 |
| Met774@CB--FMM@C31 | 59 |  |  | 57 |
| Met774@CB--FMM@C32 |  |  | 52 | 67 |
| Met774@CG--FMM@C31 | 75 | 52 | 75 | 79 |
| Ser783@C--FMM@F | 69 | 76 | 70 | 74 |
| Ser783@CB--FMM@F |  | 70 | 54 | 60 |
| Arg784@C--FMM@F | 98 | 99 | 98 | 99 |
| Arg784@CA--FMM@F | 58 | 76 | 52 | 55 |
| Leu785@CA--FMM@F | 83 | 88 | 87 | 93 |
| Leu785@CB--FMM@F |  |  | 57 | 67 |
| Leu785@CD1--FMM@C35 | 64 | 64 | 55 | 52 |
| Leu796@C--FMM@Cl | 100 | 100 | 100 | 100 |
| Leu796@CB--FMM@Cl | 58 | 58 | 66 | 71 |
| Leu796@CD2--FMM@C28 |  |  |  | 52 |
| Val797@C--FMM@Cl | 87 | 85 | 77 | 87 |
| Val797@CA--FMM@Cl | 85 | 86 | 75 | 82 |
| Thr798@CB--FMM@Cl | 54 | 57 | 64 | 63 |
| Thr798@CG2--FMM@C33 | 84 | 74 | 79 | 78 |
| Thr798@CG2--FMM@C35 | 90 | 90 | 86 | 76 |
| Thr798@CG2--FMM@C36 | 58 |  | 52 | 59 |
| Thr798@CG2--FMM@Cl | 89 | 87 | 96 | 93 |
| Thr798@CG2--FMM@F | 100 | 95 | 100 | 100 |
| Leu800@CD1--FMM@C16 | 72 | 60 | 57 | 66 |
| Gly804@C--FMM@C15 |  |  | 62 |  |
| Gly804@CA--FMM@C15 | 85 | 80 | 88 | 85 |
| Gly804@CA--FMM@C16 | 65 |  | 55 |  |
| Cys805@CB--FMM@C10 |  |  | 74 | 74 |
| Cys805@CB--FMM@C11 |  |  | 66 | 59 |
| Cys805@CB--FMM@C12 |  |  | 53 |  |
| Cys805@CB--FMM@C9 |  | 64 | 64 | 75 |
| Arg849@CB--FMM@C6 |  | 76 |  | 85 |
| Arg849@CB--FMM@C8 |  | 74 | 66 | 80 |
| Arg849@CD--FMM@C6 |  |  |  | 56 |
| Leu852@CD1--FMM@C17 | 65 | 78 | 77 | 73 |
| Leu852@CD1--FMM@C19 | 95 | 92 | 97 | 96 |
| Leu852@CD1--FMM@C21 | 92 | 93 | 94 | 93 |
| Leu852@CD1--FMM@C39 | 75 | 87 | 82 | 79 |
| Leu852@CD2--FMM@C14 |  | 52 | 55 | 65 |
| Leu852@CD2--FMM@C17 |  |  | 55 | 53 |
| Leu852@CD2--FMM@C39 | 74 | 70 | 85 | 88 |
| Leu852@CD2--FMM@C40 | 67 | 85 | 92 | 92 |
| Thr862@CG2--FMM@C23 |  |  | 70 | 71 |
| Thr862@CG2--FMM@C24 | 68 | 99 | 100 | 100 |
| Thr862@CG2--FMM@C25 |  |  |  |  |
| Asp863@C--FMM@C30 | 82 | 74 | 83 | 85 |
| Asp863@CA--FMM@C25 |  |  |  | 53 |
| Asp863@CA--FMM@C29 | 77 |  | 75 | 75 |
| Asp863@CA--FMM@C30 | 99 | 94 | 100 | 99 |
| Asp863@CA--FMM@C31 | 73 | 64 | 75 | 73 |
| Asp863@CB--FMM@C25 | 59 | 57 | 77 | 75 |
| Asp863@CG--FMM@C25 | 67 | 94 | 84 | 75 |
| Phe864@CD1--FMM@C31 |  |  | 59 | 66 |
| Phe864@CD2--FMM@C31 | 62 | 64 | 59 | 65 |
| Phe864@CE1--FMM@C31 | 70 | 74 | 76 | 81 |
| Phe864@CE1--FMM@C32 |  | 53 | 62 | 67 |
| Phe864@CE2--FMM@C31 | 76 | 80 | 68 | 80 |
| Phe864@CE2--FMM@C32 | 92 | 94 | 89 | 93 |
| Phe864@CG--FMM@C31 |  |  |  | 57 |
| Phe864@CZ--FMM@C31 | 84 | 84 | 81 | 88 |
| Phe864@CZ--FMM@C32 | 98 | 98 | 99 | 99 |
